# Supplementary material for: Functional nanoporous graphene superlattice
Source: Nat Commun. 2024 Feb 12;15:1295. doi: 10.1038/s41467-024-45503-9 (PMC10861524; doi:10.1038/s41467-024-45503-9)
Supplement: Supplementary file 1 — Supplementary Information [file 41467_2024_45503_MOESM1_ESM.pdf]

Table of Contents

Supplementary Notes 2

Supplementary Note 1: First-principles density functional theory (DFT) simulation of element diffusion and bonding in graphene superlattice 2

Supplementary Note 2: Experimental evidence for electron domain wall in graphene superlattice 3

Supplementary Note 3: First-principles DFT simulation of electronic structure of graphene superlattice 4

Supplementary Note 4: Molecular dynamics simulation of the phonon coupling of graphene superlattice 8

Supplementary Note 5: Experimental evidence for the dielectric dispersion at lower-frequency region 9

Supplementary Note 6: Design of graphene superlattice-based device for electromagnetic–electricity conversion 10

Supplementary Note 7: The correlation between electron and phonon structures in extended applications 11

Supplementary Note 8: Calculation of electron and lattice thermal conductivity in graphene superlattice 12

Supplementary Note 9: Influence of graphene superlattice on in-plane and out-of-plane thermoelectric properties 13

Supplementary Figures 15

Supplementary Tables 35

Supplementary Table 1. Polarization frequency, maximum EM absorption efficiency, and thickness of conventional dielectric materials. 35

Supplementary Table 2. Annealing treatment conditions for element doping. 36

Supplementary References 37

Supplementary Notes

Supplementary Note 1: First-principles density functional theory (DFT) simulation of element diffusion and bonding in graphene superlattice

In this study, we employed first-principles calculations based on DFT within the Vienna Ab initio Simulation Package (VASP) to investigate the diffusion energy barrier of tellurium (Te) atoms on the surface of a graphene superlattice[1,2]. To model the system, we constructed a supercell consisting of 263 carbon (C) atoms and one Te atom. Furthermore, we created two partially overlapped square-shaped pores, each with a diameter of 1.0 nm, by selectively removing carbon atoms from the layers of pristine bilayer graphene. The overlap ratio of the pores between the layers, defined as defined by the ratio of the pore area covered by a neighboring graphene layer to the total pore area, was set to 0.5. To ensure structural stability, we saturated the edges of the pores with hydrogen (H) atoms. The exchange and correlation interactions between the electrons were described using the Perdew–Burke–Ernzerhof (PBE) function within the generalized gradient approximation[3]. Additionally, we accounted for van der Waals interactions by incorporating the semi-empirical long-range dispersion correction proposed by Grimme, known as the DFT-D2 method. The plane-wave cutoff energy was set to 520 eV to achieve accurate and converged results. Total energies and force components were converged to 1×10^{-5} eV and 1×10^{-2} eV/Å, respectively. The reciprocal space was sampled using a Monkhorst-pack k-point mesh with a point density of $2\times2\times1$.

Fig. 1i in the main text represents two selected diffusion paths, along which we determined the diffusion energy barriers using the climbing image nudged elastic band (CI-NEB) method implemented in the VASP transition state tools[4,5]. Our results indicate that the diffusion of Te atoms towards the pore edge exhibits a lower energy barrier of 0.42 eV compared to diffusion towards the framework (0.76 eV). Furthermore, we analyzed the bonding between Te and C atoms at the pore edge and in the framework. The Te–C bonding at the pore edge exhibited a significantly low negative binding energy of –7.41 eV, indicating a favorable interaction between Te and C atoms at the pore edges. In contrast, bonding of Te to C atoms in the framework yielded a positive binding energy of 0.72 eV. These findings suggest that achieving preferential element doping at the pore edges requires relatively low temperatures compared to doping within the framework[6]. In summary, our first-principles DFT calculations provide insights into the diffusion energy barriers and bonding behaviors of Te atoms in a graphene superlattice, which provide the potential for selective doping strategies and highlight the significance of pore edge effects in graphene superlattice structures.

Supplementary Note 2: Experimental evidence for electron domain wall in graphene superlattice

To gain insights into the mechanism underlying the presence of the electron domain wall, we performed an analysis of the crystal structure of the graphene superlattice using near-edge X-ray absorption fine structure (NEXAFS) characterization of the carbon atom K-edge. For comparison, we also measured pristine graphene and porous bilayer graphene with overlapped pores. The results, shown in Supplementary Fig. 10, reveal distinct features in the C K-edge spectrum of the graphene superlattice. The presence of a G peak at 285.6 eV in the C K-edge spectrum of the graphene superlattice, lower than that of pristine graphene and porous bilayer graphene, indicates partial reconstruction of sp^2 C–C bonding in the graphene lattice due to the partial overlapping of carbon atoms[11, 12]. This feature demonstrates a strong polarization dependence and suggests the formation of an electron domain wall.

Additionally, two peaks at 291.7 eV and 290.3 eV in the graphene superlattice spectrum are attributed to the $1s \rightarrow \sigma^*$ transition, indicating the presence of two types of disorders[13,14]. These disorders arise from in-plane strain and non-graphitized carbon-based bonds at the edges. Interestingly, the absence of a peak caused by lattice strain in both pristine graphene and porous bilayer graphene with overlapped pores suggests that lattice strain has been overlooked[15]. The combination of in-plane lattice strain and polarized C–C bonds in the graphene lattice collectively induces the formation of the electron domain wall. Furthermore, we observed that the G peak in the C K-edge spectrum of Te-doped graphene superlattice is further shifted to the left, and the intensity of the two disorder peaks is increased compared to the graphene superlattice. This indicates an enhanced in-plane strain and an increased intensity of polarized C–C bonding in the graphene lattice[16]. Overall, these findings provide experimental evidence for the presence of an electron domain wall in the graphene superlattice and shed light on its underlying structural characteristics.

Supplementary Note 3: First-principles DFT simulation of electronic structure of graphene superlattice

Here we utilized first-principles DFT to simulate the band structure of graphene. Our simulation process involved several steps, including crystal model design and optimization, building of a static self-consistent field, and calculation of the density of states[17,18]. All calculations were performed using VASP-6.1.0 with the PBE function under the generalized gradient approximation. The DFT framework with D3 dispersion correction was combined with the projector augmented wave, and a plane wave cutoff energy of 500 eV was set[19]. The K-mesh for calculating the band structures of the samples was generated using vaspkit-1.2.3 with a line-mode consisting of 20 points. To prevent artificial interactions between periodic images, a 20 Å-thick vacuum layer was introduced perpendicular to the graphene sheet. The structures were relaxed until the residual forces on the atoms decreased to below 0.05 eV/Å. Phonon structures of graphene were calculated using the finite displacement method combined with phononpy-2.11[20].

In our study, we considered various factors, such as pore overlap ratio, doping amount and types, as well as other in-plane defect engineering methods, to comprehensively analyze their effects on the electronic band structure of graphene.

(i) Effect of nanopore overlap ratio

To investigate the impact of the pore overlap ratio on the electronic structure, we performed calculations on doped porous graphene systems with square-shaped nanopores with a diameter of 1.0 nm overlapping at different ratios: complete overlap (1), partial overlap (0.5), and no overlap (0). In these systems, the carbon atoms at the pore edge of the nanopore were saturated with an equal number of Te atoms. Pristine bilayer graphene was also included in the calculations for comparison. The electronic properties of graphene are primarily influenced by the behavior of the conduction and valence bands near the Fermi level. Therefore, these bands were analyzed due to their crucial role in determining the electronic characteristics of graphene. As shown in Supplementary Fig. 16, pristine graphene exhibits distinct conduction and valence bands with a cone-shaped structure that converges at the Dirac point. This cone-like dispersion signifies a strong linear relationship between electron energy and kinetic energy. However, there is no significant electronic density of states observed near the Fermi level.

When fully overlapped pores are introduced to bilayer graphene (the overlap ratio is 1), the Dirac point splits, resulting in the emergence of an open bandgap with a magnitude of 0.25 eV. Importantly, the band splitting does not significantly affect the electronic density of states near the Fermi level. With partial overlapping of the nanopores in bilayer graphene (the overlap ratio is 0.5), the band exhibits a remarkable flattening near the Fermi level, characterized by negligible band dispersion (less than 1 meV). This flat band structure arises from the weak dispersion relation of kinetic energy due to the periodic electronic reconstruction caused by atomic stress between the overlapped and exposed carbon regions[21,22]. This reconstruction leads to the formation of electron domain walls that confine Fermi electrons on either side, resulting in an equipotential Fermi surface and energy barriers with neighboring surfaces. The electron trapping effect leads to a closer proximity of valence electrons in the graphene

superlattice to the Fermi level, resulting in the formation of multiple van Hoff singularities[22,23], as demonstrated by the electron density distribution (Supplementary Fig. 12). When the nanopore is not overlapped (the overlap ratio is 0), the band shows a noticeable degree of dispersion (greater than 50 meV) compared to partially overlapping nanopores in bilayer graphene. However, it remains significantly weaker than the dispersion observed in pristine graphene and porous graphene with fully overlapped nanopores. It can be explained that in the case of completely non-overlapping nanopores, the graphene structure exhibits an increased area of exposed regions, in the form of monolayer graphene. This enlarged area provides a platform for the release of interfacial strain that is generated between the overlapped regions of bilayer graphene and the exposed regions. Consequently, the electron domain wall at the interface is weakened, leading to a reduced ability to trap electrons. This weakening of the electron domain wall has a direct impact on the dispersion of the bands and limits their proximity to the Fermi level. As a result, lower electronic density of states near the Fermi level and non-negligible band dispersion are observed.

(ii) Effect of doping element concentration

In the case of doped graphene with partially overlapped nanopores (the overlap ratio is 0.5), we conducted further investigations to examine the influence of varying amounts of Te doping on the pore edges. Specifically, the carbon atoms at the edge of one pore were saturated with 2–10 Te atoms, while the remaining atoms were bonded with H. In the case of the graphene superlattice without Te doping, the pore edge was saturated with –OH, –COOH, and –OH bonds, which aligns with the graphene superlattice without edged doping.

As depicted in Supplementary Fig. 13, the band gradually turns to a flat shape as the Te atom content at the edges increases. When the number of Te atoms at the pore edge exceeds 8, the band dispersion becomes negligible (less than 1.0 meV). This observation indicates that high levels of Te doping at the pore edge effectively suppress the dispersion relationship between electron energy and kinetic energy, thereby contributing to the electron trapping effect[24]. Additionally, an increase in Te content leads to an upward shift of the valence band, resulting in the formation of one or multiple van Hoff singularities and a significant increase in the electronic density of states near the Fermi level. As the Te content further increases, the band dispersion becomes negligible, and the electron confinement becomes stronger, preventing the electrons from shifting further upward and confining them near the Fermi surface. Instead, the valence band located below the uppermost band continues to shift upward, leading to their overlapping and resulting in the merging of Fermi electrons with one strong van Hoff singularity near the Fermi level (exceeding 300 eV^{-1}).

(iii) Effect of doping element types

In the case of bilayer graphene with partially overlapped nanopores (overlap ratio of 0.5), we conducted additional investigations to analyze the impact of different doping element types at the pore edges. Specifically, we saturated the carbon atoms at one pore edge with eight representative atoms of nitrogen (N), phosphorus (P), or sulfur (S), while the remaining atoms were bonded with H. The results depicted in Supplementary Fig. 14 demonstrate distinct effects of substituting the dopant element. When N is used as the dopant, a remarkable band dispersion exceeding 50 meV is observed. However, there

is a low electronic density of states near the Fermi level, indicating a weak electron trapping effect and a lack of significant upward shift in the valence band. As we replace N with heavier elements such as P and S, the band dispersion gradually decreases with increasing atomic number. Additionally, the electronic density of states near the Fermi level experiences a notable increase. In the case of S substitution, two continuous van Hoff singularities emerge near the Fermi level with peak intensities surpassing 140 eV^{-1}. These intensities are approximately six times higher than that of N-doped graphene and three times higher than that of P-doped graphene, indicating a substantial upward shift in the valence band and pronounced merging of bands[25].

The observed changes can be attributed to the energy difference between the bonding orbitals of the dopant atom (e.g., N for *2p,* S and P for *3p*) and the *2p* orbital of the edge carbon. As the atomic number increases, this energy difference gradually enhances polarization. The enhanced polarization, facilitated by the orbital–spin coupling, leads to band splitting near the Fermi level and promotes an upward shift in the valence band[26,27]. Consequently, it increases the number of valence bands near the Fermi level, promotes the formation of multiple van Hoff singularities, and intensifies their strengths[28]. The increased electronic density of states contributes to band flattening and enhances the electron trapping effect. However, when the energy difference exceeds a certain threshold, such as in the case of the *6p* orbital of Te and the *2p* orbital of C, the covalent bonds between the dopant atoms and the edge carbon can transform into coordination bonds[29]. This transformation weakens the strength of orbital–spin coupling due to weak overlapping of orbital wave functions[30]. Consequently, the intensities of the peaks and the electron trapping effect is somewhat diminished.

(iv) Effect of nanopore shape

We conducted further investigations to explore the influence of nanopore shape on bilayer graphene by considering partially overlapping circular nanopores. The overlap ratio was set at 0.5, and the edges of the pores were saturated with 8 Te atoms, following the same configuration as the Te-doped graphene superlattice mentioned earlier. As shown in Supplementary Fig. 15, the presence of circular nanopores leads to the splitting of the Dirac point, resulting in the emergence of a narrow band gap of approximately 0.1 eV. The underlying reason behind this behavior may lie in the structural characteristics of bilayer graphene with partially overlapping circular nanopores. It exhibits periodicity, overlapping, and exposed carbon atoms. However, the exposed region forms a curved nanoribbon with wider ends and a narrower waist, which introduces strain into the system. The generation of such strain disrupts the stress difference at the interface between the exposed and overlapped carbon atoms, making it challenging to effectively construct electron domain walls. Without electron domain walls, the confinement of electrons is hindered, and the generation of significant energy splitting is impeded. Consequently, there is a lack of strong electron coupling, leading to a weak band dispersion[31]. In contrast to the flat band structure in graphene with square nanopores, the presence of circular nanopores in bilayer graphene with partial overlap does not result in a flat band structure. Instead, it exhibits a narrow band gap without significant electronic density of states near the Fermi level.

(v) Effect of in-plane defect engineering

Defect engineering is a widely used technique for manipulating the band structure of graphene,

allowing precise control over its electronic properties. This approach involves introducing defects such as vacancies and specific element doping. Additionally, the electronic structure of graphene can be customized by creating graphene nanoribbons with controlled dimensions. Here we examined three types of defected graphene: Te-doped pristine graphene, graphene with vacancy defects (e.g., a removed carbon atom), and an armchair graphene nanoribbon measuring 2.0 nm in length and 0.5 nm in width. The edges of the graphene nanoribbon were saturated with H atoms. As depicted in Supplementary Fig. 16, all three types of graphene demonstrate distinct dispersion behavior in their band structures, accompanied by varying degrees of Dirac point splitting, ultimately leading to the formation of bandgaps. Furthermore, there is no significant electronic density of states observed near the Fermi level. These observations can be attributed to the absence of periodic electron domain walls within the graphene layers, resulting in a negligible electron coupling effect[32,33].

Supplementary Note 4: Molecular dynamics simulation of the phonon coupling of graphene superlattice

To evaluate the degree of phonon coupling in the graphene superlattice and assess the compatibility of the newly generated low-frequency phonons, molecular dynamics simulations were conducted using the open-source code large-scale atomic/molecular massively parallel simulator (LAMMPS)[34-36]. The computational model was designed to align with experimental data, and square pores with an overlap ratio of 0.5 was chosen for the graphene superlattice system. The square nanopores in the simulation had a diameter of 7.0 nm, and the width between nanopores on each atomic layer was set to 1.0 nm. The simulation process involved initial energy minimization, followed by relaxation under the constant pressure and temperature (NPT) ensemble at 300 K for 50 ps, with a time step of 1 fs. Subsequently, the simulation continued at 300 K for an additional 100 ps under the constant volume and temperature (NVT) ensemble. During the last 50 ps of the simulation, the velocities of the carbon atoms of interest were sampled every 5 fs to calculate the vibrational density of states[37-39].

To accurately model the interactions between the exposed and overlapped carbon atoms in the graphene superlattice, the adaptive intermolecular reactive empirical bond order (AIREBO) potential was employed[40]. The classification of carbon atoms within the system was based on their proximity to the pore, where carbon atoms within a distance smaller than 10 Å were identified as pore edge carbon atoms, while carbon atoms beyond this distance were considered as carbon atoms within the framework. The vibrational density of states, denoted as $P(\omega)$, was calculated as[41]:

$$\mathrm{P}(\omega)=\frac{1}{\sqrt{2\pi}}\int_0^{\infty} \mathrm{e}^{i\omega t}\left\langle\sum_{\mathrm{j}=1}^{\mathrm{n}} v_j(t)v_j(0)\right\rangle \mathrm{d}\omega \tag{S1}$$

where ω and v_j(t) represent the angular frequency and velocity of carbon atom j at time t, respectively. The ensemble average in Equation S1 was approximated by a time average computed over a period of 50 ps once the system reached equilibrium. The coupling degree between any two calculated vibrational density of states (P(ω)) can be determined by[42]:

$$\text{Phonon coupling}=\left(\int \sqrt{P_1(\omega)P_2(\omega)}\,\mathrm{d}\omega\right)^2 \Big/ \left(\int P_1(\omega)\mathrm{d}\,\omega \int P_2\,(\omega)\mathrm{d}\omega\right) \tag{S2}$$

The results depicted in Supplementary Fig. 17 demonstrate that the phonon coupling between the carbon atoms at the pore edge and in the framework is relatively weak, with a value as low as 0.71. Similarly, the coupling degree between the overlapped carbon atom and the exposed carbon atom is even lower, reaching a value as low as 0.51. These findings indicate poor compatibility among the newly generated phonons, resulting in increased scattering between them. Consequently, the phonon thermal conductivity is significantly reduced, leading to a decrease in the overall thermal conductivity.

Supplementary Note 5: Experimental evidence for the dielectric dispersion at lower-frequency region

The permittivity of graphene as a function of EM wave frequency is shown in Supplementary Fig. 20. In the case of pristine bilayer graphene, both the real part (ε') and the imaginary part (ε'') of the permittivity decrease as the frequency increases from 1 GHz to 5 GHz. However, for element-doped graphene superlattice, a distinct behavior is observed. Specifically, there is a sharp decrease in ε' and a remarkable resonance peak in ε" within the same frequency range. The presence of a resonance peak in ε'' indicates a frequency-dependent response, which can be attributed to the phenomenon of polarization relaxation[43,44]. According to the classical Debye theory, polarization relaxation gives rise to a semicircular relationship between ε' and ε'' known as the Cole–Cole semicircle. The relative complex permittivity can be expressed as[45,46]:

$$\varepsilon_r=\varepsilon_\infty+\frac{\varepsilon_s-\varepsilon_\infty}{1+i2\pi f\tau}=\varepsilon'-j\varepsilon'' \tag{S3}$$

where ε_s represents the static permittivity, ε_∞ is the relative dielectric permittivity at the high-frequency limit, f is the frequency, i is the imaginary unit, and τ is the dipole relaxation time. By separating the real and imaginary parts, we will obtain[47,48]:

$$\varepsilon'=\varepsilon_\infty+\frac{\varepsilon_s-\varepsilon_\infty}{1+(2\pi f)^2\tau^2} \tag{S4}$$

$$\varepsilon''=\varepsilon_\infty+\frac{2\pi f\tau(\varepsilon_s-\varepsilon_\infty)}{1+(2\pi f)^2\tau^2} \tag{S5}$$

$$(\varepsilon'-\varepsilon_\infty)^2+(\varepsilon'')^2=(\varepsilon_s-\varepsilon_\infty)^2 \tag{S6}$$

Based on Equation S6, which describes the relationship between ε' and ε'', each Cole–Cole semicircle corresponds to a Debye relaxation process. In the case of doped graphene superlattices, the presence of Cole–Cole semicircles (Supplementary Fig. 21) indicates the occurrence of dipole relaxation polarization behavior in the low-frequency range, primarily attributed to the presence of electron domain walls[49,50]. It is important to note that achieving low-frequency polarization relaxation remains a significant challenge for current dielectric materials[51-75], as illustrated in Supplementary Table 1.

Supplementary Note 6: Design of graphene superlattice-based device for electromagnetic–electricity conversion

The graphene superlattice-based device used in this study involves two main steps for harvesting electromagnetic (EM) energy and converting it into direct current (DC) electricity. First, the graphene superlattice-based device captures and converts EM energy into oscillating alternating current (AC) electricity. This operation is based on the principles of EM induction and radiation[76]. When an EM wave interacts with the graphene superlattice-based device, the alternating electric and magnetic fields induce an oscillating current in the graphene superlattice. Second, a rectifier is employed to convert the oscillating AC into DC[77]. The rectifier utilizes the non-linear characteristic of a diode, allowing current to flow in only one direction and effectively rectifying the oscillating current[78].

The design of the graphene superlattice-based device involves two main components:

(i) Graphene superlattice-based device

The optimal geometric shape suitable for multiple lower-frequency bands is determined using Computer Simulation Technology Microwave Studio software. The specific thickness (20 μm) and electrical conductivity (1,064 S/cm) of the graphene superlattice film are taken into consideration. Supplementary Fig. 23 illustrates the detailed geometric shapes and specific dimensions of the graphene superlattice-based device. To fabricate the graphene superlattice-based device, the graphene superlattice film is attached to a polydimethylsiloxane (PDMS) substrate with a thickness of 200 μm. Laser direct molding is then employed to shape the graphene superlattice film into the simulated geometric shapes, achieving the desired structure.

(ii) Rectifier

The rectifier consists of three parallel rectification branches to accommodate multiple low-frequency bands. Each branch includes a series connection of a diode, a DC-pass filter, a diode front-end transmission line, and an impedance matching network. The detailed geometric shapes and specific dimensions of the rectifier are calculated using the Advanced Design System software, as shown in Supplementary Fig. 24. The rectifier is manufactured by first attaching a 35 μm-thick Cu foil onto a dielectric substrate called Deriod 5880, which has dimensions of 54 mm in length, 42 mm in width, and 0.79 mm in thickness. The dielectric substrate has a relative permittivity of 2.2 and a tangent loss of approximately 0.0009. The Cu foil is then shaped into the desired current mode using high-frequency circular pressing technology.

By combining the graphene superlattice-based device for capturing and converting EM waves and the rectifier for rectifying the oscillating current, the integrated graphene superlattice-based device enables the efficient conversion of EM energy into stable DC electricity.

Supplementary Note 7: The correlation between electron and phonon structures in extended applications

(i) Connection of electron structure with EM modulation and luminescence performance:

Observations of the graphene superlattice's band indicate multiple van Hoff singularities near the Fermi level (Fig. 2a). These singularities stem from electron domains that split the Fermi surface, creating new Fermi surfaces with differing energy levels. This property enables the graphene superlattice to harness external electric fields to excite Fermi electrons, consequently enhancing conductivity and linearly influencing the permittivity. The linear increase in permittivity indicates the film's suitability for EM wave transmission, absorption, and shielding as it progressively rises, suggesting EM switchability (Fig. 4a). The increased energy level splitting and potential differences across the electron domain wall lead to the separation of electrons and holes, producing a notable photoluminescence effect in the visible light wavelength range (400–700 nm), distinct to the graphene superlattice and not observed in pristine graphene (Fig. 4b).

(ii) Connection of phonon structure with reduced thermal conductivity:

Upon examining the impact of the superlattice structure on the graphene phonon structure, it becomes apparent that pristine graphene predominantly features atom-propagated phonons at a frequency of 50 THz (Fig. 2c). However, in bilayer graphene superlattices, the periodic overlap of carbon atoms hinders harmonic vibrations, creating distinct phonon modes in the overlapped and exposed regions. This unique transmission of phonon clusters results in significant coherent interference and elastic scattering, weakening the energy of the phonon clusters. This generates a series of continuous standing waves in the low-frequency range of 10–30 THz, substantially reducing the phonon mean free path. Consequently, this reduction leads to notable localization and a decline in thermal infrared emission (Fig. 4c and Supplementary Fig. 28).

(iii) Connection of electron–phonon Interaction with thermoelectric behavior:

The localization of low-frequency phonons and the electron trapping effect causes a strong electron–phonon coupling. As a result, electrons absorb phonons and scatter to excite new phonons, initiating a pronounced phonon drag effect. This effect significantly impacts the trajectories and transport properties of electrons within the graphene superlattice. This substantial phonon drag positively influences the Seebeck coefficient (Fig. 4d). The amplified Seebeck coefficient, combined with reduced thermal conductivity, results in a nearly two orders of magnitude higher ZT value compared to pristine graphene, showcasing its exceptional promise for thermoelectric applications (Fig. 4f).

Supplementary Note 8: Calculation of electron and lattice thermal conductivity in graphene superlattice

To comprehensively analyze the thermal conductivity (κ_T) mechanism in the device, a calculation based on the Widemann–Franz relationship allows the determination of lattice (κ_L) and electron thermal conductivity (κ_e)[79]:

$$\kappa_T = \kappa_e + \kappa_L \tag{S7}$$

$$\kappa_e = \sigma \times L \times T \tag{S8}$$

where L represents the Lorenz number (in units of 10^{-8} WΩK^{-2}), The Lorenz number L has a proportional relationship with the Seebeck coefficient α, outlined as[80]:

$$L = 1.5 + \mathrm{e}^{-\frac{|\alpha|}{116}} \tag{S9}$$

Utilizing the above equations, the in-plane values for κ_e and κ_L can be determined at increasing temperatures. Upon analysis, it becomes apparent that in-plane κ_L predominantly governs the overall thermal conductivity (Supplementary Fig. 27). In comparison to pristine graphene, a significant reduction in in-plane κ_L by approximately 98% stands out as the primary factor contributing to the observed diminished thermal conductivity. This substantial decrease in in-plane κ_L is intricately linked to alterations in the phonon structure. Pristine graphene mainly exhibits atom-propagated phonons oscillating at a frequency of 50 THz, as depicted in Fig. 2c. This behavior stems from the harmonic oscillation inherent in the atomic lattice. In contrast, the periodic overlap of carbon atoms in bilayer graphene superlattices suppresses these harmonic vibrations, resulting in the emergence of distinct phonon modes in the overlapped and exposed regions. The transmission of these phonon clusters exhibits considerable coherent interference and elastic scattering. Consequently, phonon cluster energy weakens and a series of continuous standing waves arise in the low-frequency range of 10–30 THz, while the original high-frequency phonons diminish. These alterations are directly attributed to the observed reduction in in-plane thermal conductivity.

Supplementary Note 9: Influence of graphene superlattice on in-plane and out-of-plane thermoelectric properties

To assess the influence of the graphene superlattice on both in-plane and out-of-plane directions, we conducted measurements of various thermoelectric properties, including electrical conductivity, Seebeck coefficient, and thermal conductivity (Supplementary Figs. 29 and 30).

i) In-plane direction:

For a comprehensive evaluation of the in-plane thermoelectric characteristics, we employed the vacuum filtration technique to fabricate films with a specific orientation. Strips measuring 6 mm × 4 mm were crafted from these films. Approximately 15 of these strips, each 6 mm in length and 4 mm in width, were assembled to form a 3 mm-thick film. Electrodes were attached at both ends to facilitate measurements of in-plane electrical conductivity and the Seebeck coefficient along the film's length. To evaluate in-plane thermal conductivity, the filtered film was precisely sectioned into strips measuring 15 mm × 5 mm. About fifty of these strips were stacked to create a film approximately 10 mm thick, and in-plane thermal conductivity was measured with heat flow directed along the film's length.

ii) Out-of-plane direction:

For measurements of out-of-plane electrical conductivity and the Seebeck coefficient, we stacked around a dozen filtered films to form a layered film with dimensions of 6 mm in length, 4 mm in width, and 3 mm in thickness, tailored to our equipment's testing parameters. The evaluation focused on the out-of-plane Seebeck coefficient and electrical conductivity orthogonal to the stacked layers. In assessing thermal conductivity, we utilized this film, comprising several ~0.2 mm thick layers and achieving an overall thickness of 5 mm. The out-of-plane thermal evaluation process ensured heat flow directed orthogonally through the plane of the film.

The results are summarized below:

i) In-plane performance:

In-plane thermal conductivity for the superlattice ranged between 6.4–4.4 W/mK, representing only 3.6–2.6% of pristine graphene's values. The Seebeck coefficient exhibited a remarkable increase of 710–560% compared to pristine graphene, while the electrical conductivity decreased by only 23.1% to 15.8%. The in-plane ZT value for the superlattice peaked at 0.33 at 500 K, significantly surpassing that of pristine graphene.

ii) Out-of-plane performance:

Out-of-plane thermal conductivity measured between 0.96 W/mK to 0.24 W/mK, with electrical conductivity recorded at 5.9–3.0 S/cm and the Seebeck coefficient varying between –7.5 μV/K to –12.0 μV/K. These measurements closely aligned with those of pristine graphene, resulting in out-of-plane ZT values that were 3 to 4 orders of magnitude lower compared to the in-plane values. We noted that the in-plane Seebeck coefficient of the graphene superlattice exceeds that in the out-of-plane direction, by a factor of approximately 8.8 to 6.3 in the temperature range of 300–500 K. This difference is attributed to the higher density of partially overlapped nanopores between graphene layers, facilitating the formation of periodic electron domains along the in-plane direction. In contrast, in the out-of-plane

direction, a larger energy barrier, with insulator-like characteristics, is present, leading to a weaker Seebeck effect.

Our investigation into the remarkable in-plane thermoelectric enhancement and relatively limited effects in the out-of-plane direction focused on pivotal structural modifications. These include the strategic creation of nanopores and selective edge doping within the plane, differing from conventional methods that alter interlayer interactions. Our synthetic method significantly modified in-plane electron and phonon structures, creating phenomena such as electron traps, coherent interference, elastic phonon scattering, and robust electron–phonon coupling. These collective alterations notably elevated the in-plane *ZT* value. While exploring interlayer dynamics, adjustments such as nanopore introduction or element doping had limited impact on fundamental interlayer forces, resulting in only a slight improvement in out-of-plane thermoelectric properties when compared to pristine graphene.

Supplementary Figures

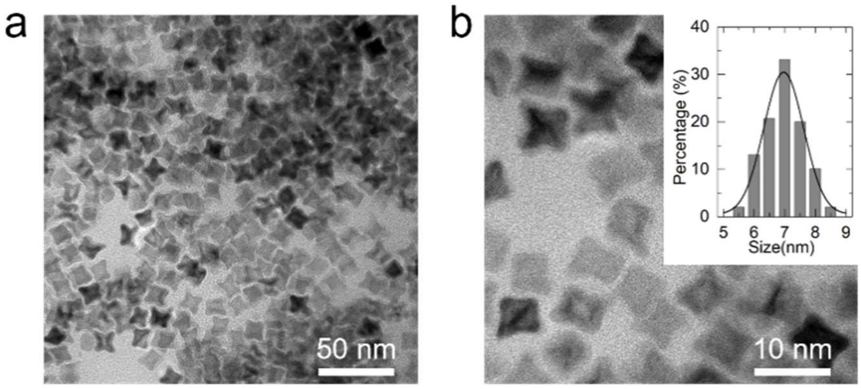

Supplementary Fig. 1 – *In-situ* growth of cubic Fe_3O_4 nanoparticles on graphene. a, b, Representative transmission electron microscope (TEM) images of cubic Fe_3O_4 nanoparticles. Inset in (b) shows the size distribution of Fe_3O_4 nanoparticles.

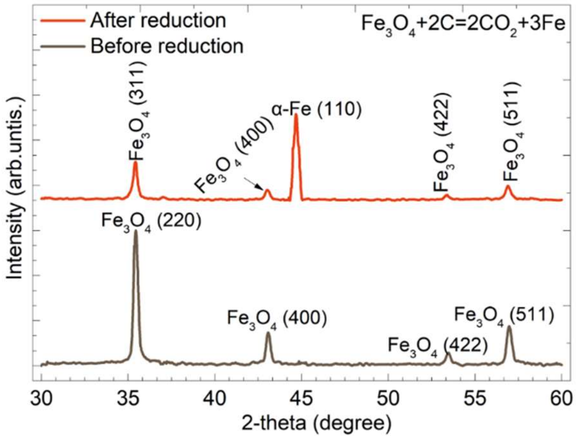

Supplementary Fig. 2 – X-ray diffraction (XRD) patterns revealing the crystal phase and composition evolution of cubic Fe_3O_4 nanoparticle-coated graphene upon annealing. The appearance of a diffraction peak at $2\theta = 44.4^{\circ}$ in the annealed Fe_3O_4 nanoparticle-coated graphene is attributed to the (110) crystal plane of α-Fe.

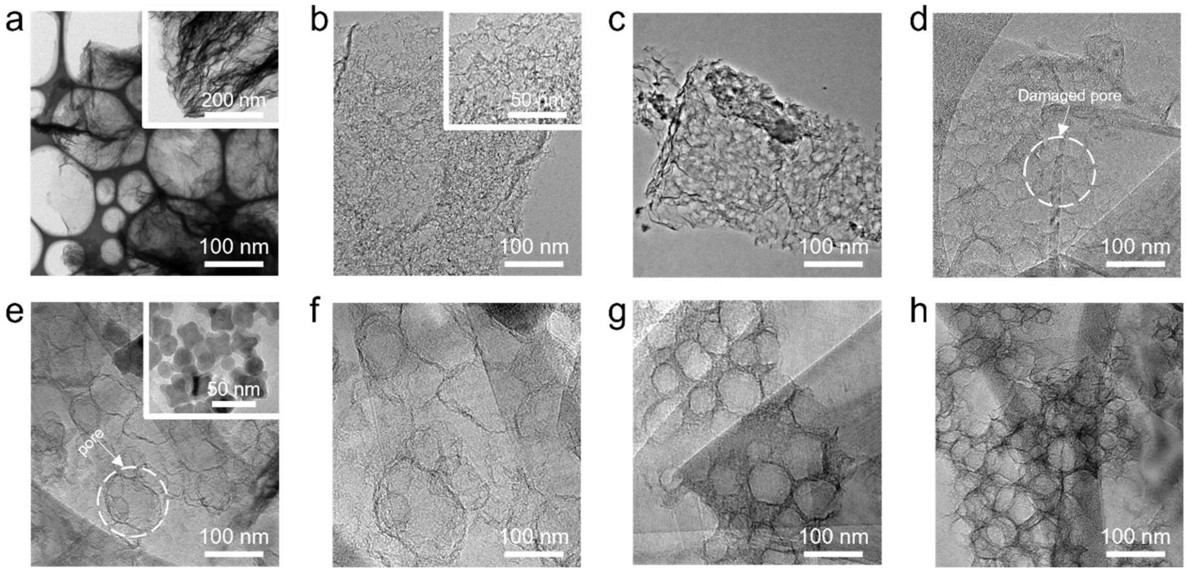

Supplementary Fig. 3 – Morphology of reduced graphene oxide and porous graphene. a-h, Representative TEM images of (a) wrinkled pristine reduced graphene oxide, (b) porous reduced graphene oxide nanosheets obtained by annealing cubic Fe_3O_4 nanoparticle-coated reduced graphene oxide, (c, d) porous graphene annealed at 650 °C for (c) 15 minutes and (d) 1 hour, (e, f) porous graphene annealed at obtained by incorporating an excessive quantity of cubic Fe_3O_4 nanoparticles (the loading amount of Fe_3O_4 nanoparticles is approximately 1.2 times greater than the amount used during the production of graphene superlattice), (g, h) porous graphene obtained by annealed at 1,000 °C and (g) 800 °C for 30 minutes. The observations reveal that insufficient annealing time results in incomplete reduction between the cubic Fe_3O_4 nanoparticle template and carbon atoms, hindering the formation of monodisperse nanopores on the graphene surface. Prolonged reaction time leads to excessive reduction and polydisperse and distorted pores, as the cubic Fe_3O_4 nanoparticle template continues to react with carbon on the second layer. High annealing temperature or excessive loading amounts causes cubic Fe_3O_4 nanoparticle template recrystallization and agglomeration, resulting in polydisperse and distorted pores. Precise control of reaction time, temperature and loaded amount is crucial for achieving monodisperse, square nanopores.

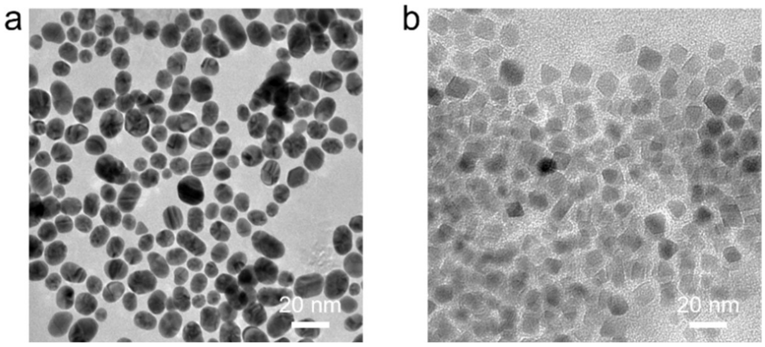

Supplementary Fig. 4 – Morphology of CoO and NiO nanoparticles. a, b, TEM images revealing irregular ellipsoidal and polyhedral (a) CoO and (b) NiO nanoparticles, formed through complete substitution of $Fe(acac)_3$ with $Co(acac)_2$ and $Ni(acac)_2$, respectively.

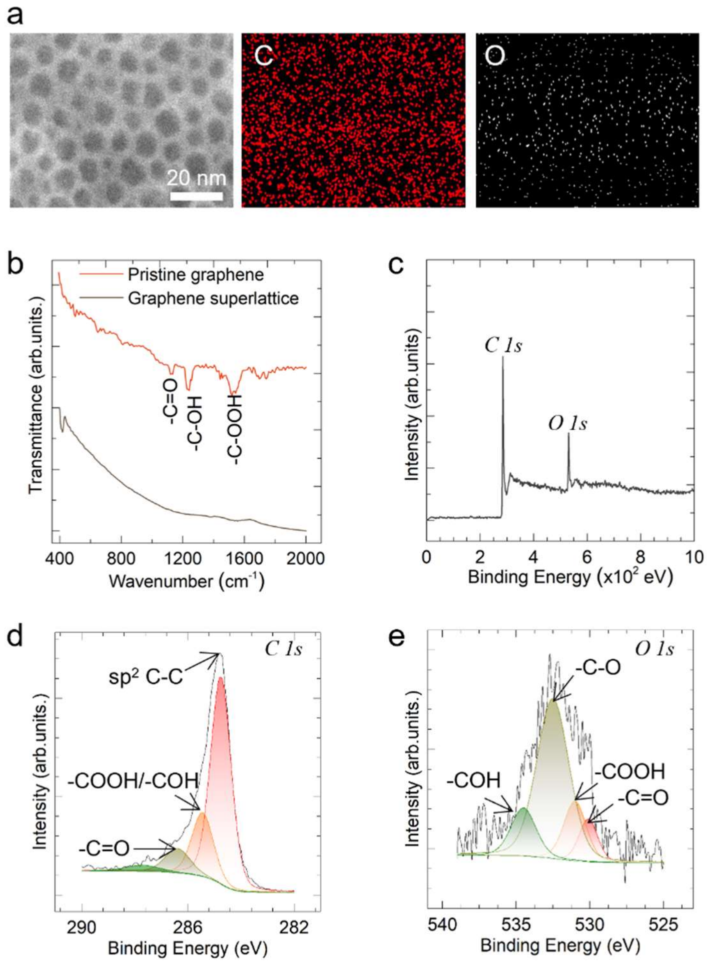

Supplementary Fig. 5 – Composition and crystal structure of graphene superlattice. a, TEM element mapping of graphene superlattice. **b,** FT-IR spectra of graphene superlattice and pristine bilayer graphene. **c,** X-ray photoelectric energy survey spectrum of graphene superlattice. **d, e,** X-ray photoelectron energy spectrum (XPS) of *C 1s* and *O 1s* spectra of graphene superlattice.

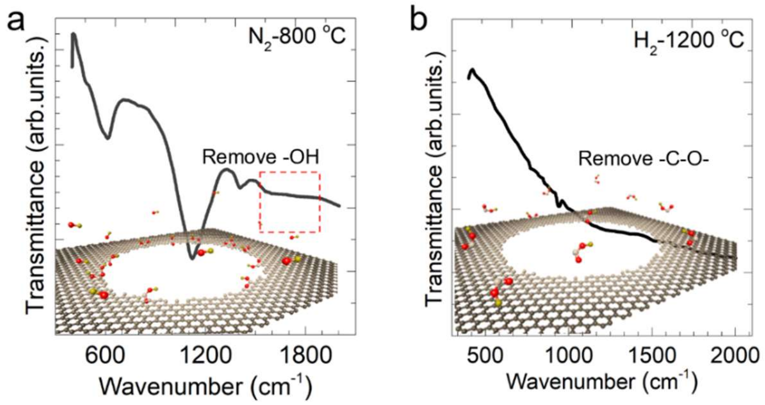

Supplementary Fig. 6 – Fourier Transform Infrared Spectra (FT-IR) of annealed graphene superlattice. a, b, The FT-IR spectra of graphene superlattice annealed at 800 °C in nitrogen and 1,200 °C in hydrogen. Upon annealing, the peaks attributed to –C–O– and –C=O bonds disappear from the spectra, indicating the removal or decomposition of oxygen-containing functional groups.

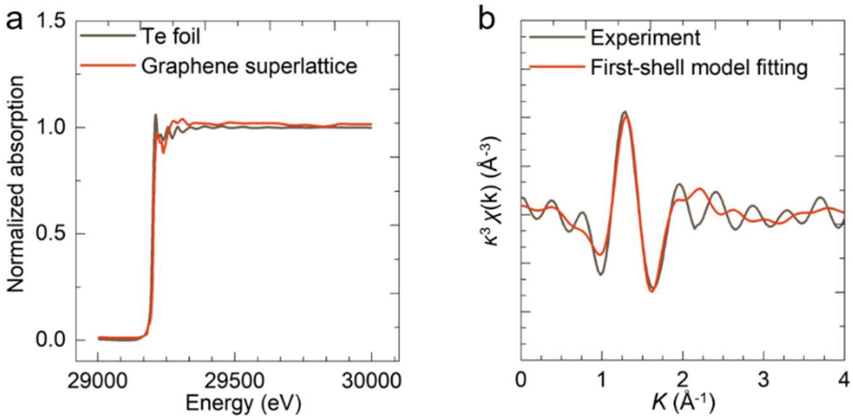

Supplementary Fig. 7 – Extended X-ray absorption fine structure (EXAFS) analysis of graphene superlattice. a, Normalized Te K-edge EXAFS spectra of Te foil and Te-doped graphene superlattice. **b,** First-shell model EXAFS fit of the Fourier transform at Te K-edge for Te-doped graphene superlattice. The EXAFS data were analyzed using the Athena modules within the IFEFFIT software packages. The preprocessing of the raw EXAFS spectra involved subtracting the pre-edge background absorption and normalizing the post-edge to a unity value. The data were then transformed from energy space to photoelectron momentum vector k space, employing a k^3-weighted method to compensate for oscillatory decay. Fourier transformation of the k^3-weighted $\chi(k)$ function with a Hanning window was performed to generate the R-space spectra, representing bond distances (R) in real space. The Artemis module was utilized for extracting local structural information through least-square curve parameter fitting, where theoretical models were fitted to the experimental data to determine the characteristic features of the local atomic arrangement.

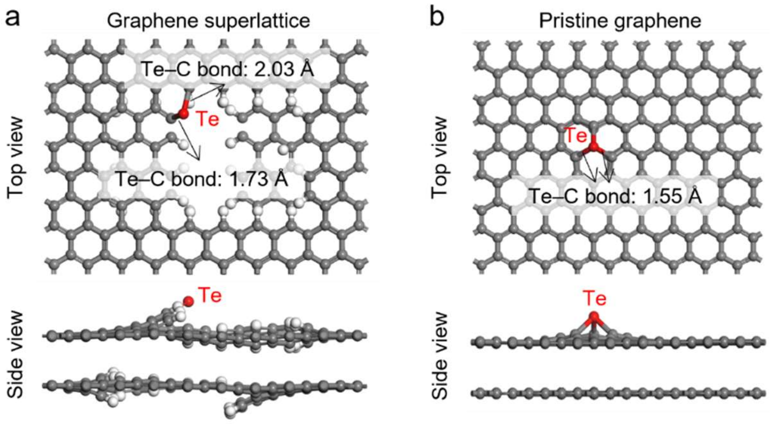

Supplementary Fig. 8 – DFT calculation of Te–carbon bond length in Te-doped graphene and graphene superlattice. a, Te-doped graphene superlattice. **b,** Te-doped pristine graphene. The computational results reveal that Te doping at the pore edge of a graphene superlattice leads to the formation of Te-C bonds with lengths of 1.73 Å and 2.03 Å, consistent with experimental observations. In contrast, Te incorporation into the graphene framework results in Te bonding to three neighboring carbon atoms with a uniform bond length of 1.53 Å.

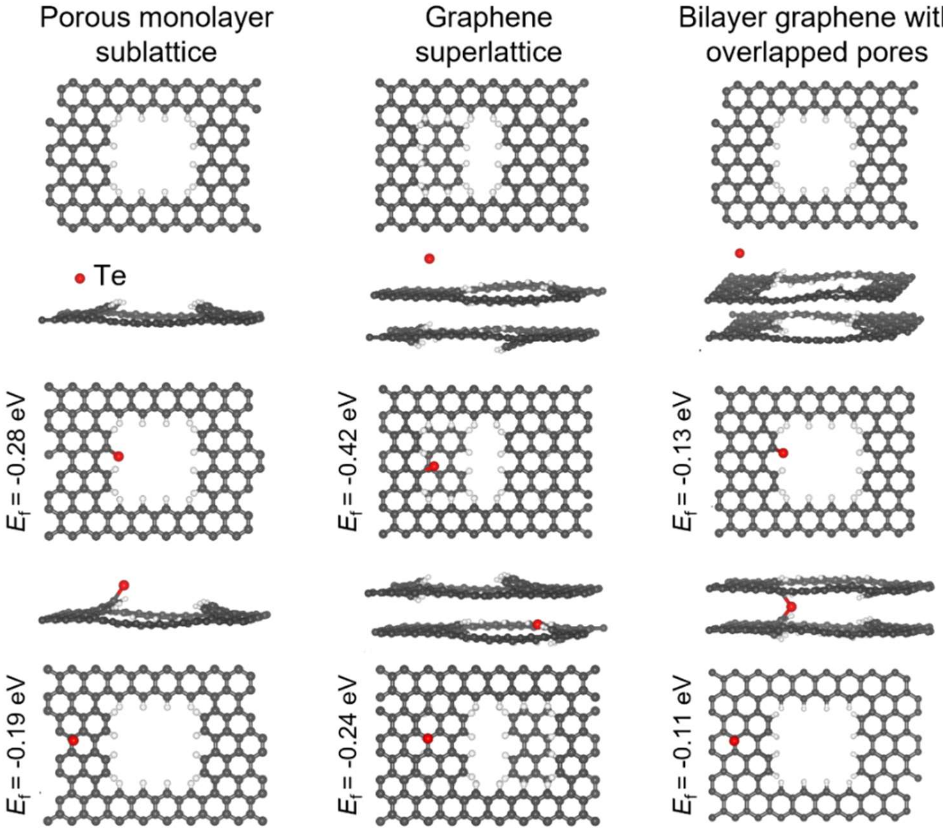

Supplementary Fig. 9 – DFT simulations of Te atoms bonded to carbon atom in the framework and at the edge. We conducted DFT simulations to investigate the binding energy (E_f) when Te atoms are bonded to carbon atoms in different configurations. Three models were considered: i) porous monolayer sublattice, ii) graphene superlattice, and iii) bilayer graphene with completely overlapped pores. For each model, we calculated E_f associated with Te doping in the graphene framework and at the pore edge. The results reveal that E_f of Te atoms bonded to carbon atoms at the pore edge is lower compared to doping in the framework. This finding indicates that successful incorporation of Te atoms through edge-doping requires a relatively lower temperature.

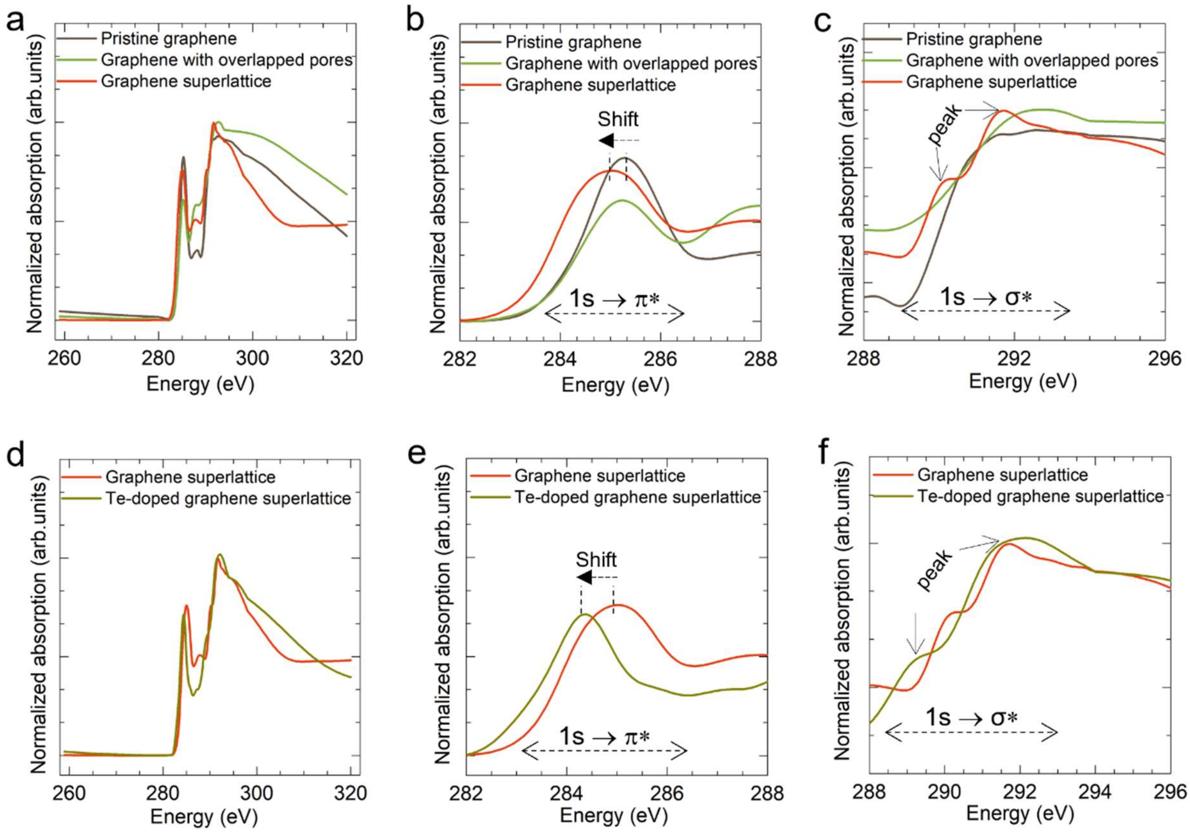

Supplementary Fig. 10 – Analysis of crystal structure of graphene superlattice. a-c, Near-edge X-ray absorption fine structure (NEXAFS) of C K-edge of pristine graphene, bilayer graphene with complete overlapped nanopores, and graphene superlattice. **d-f,** NEXAFS of C K-edge of graphene superlattice and Te-doped graphene superlattice.

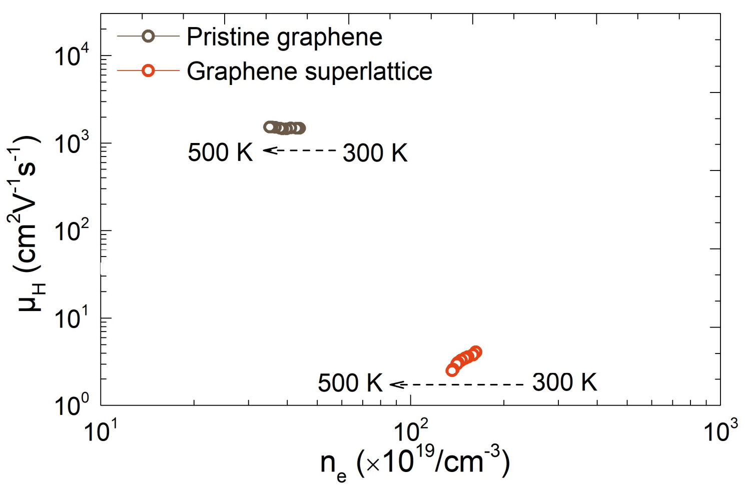

Supplementary Fig. 11 – Temperature-dependent carrier mobility of pristine graphene and porous graphene. Temperature-dependent carrier mobility (μ_H) and Hall carrier density (n_e) of pristine bilayer graphene and graphene superlattice.

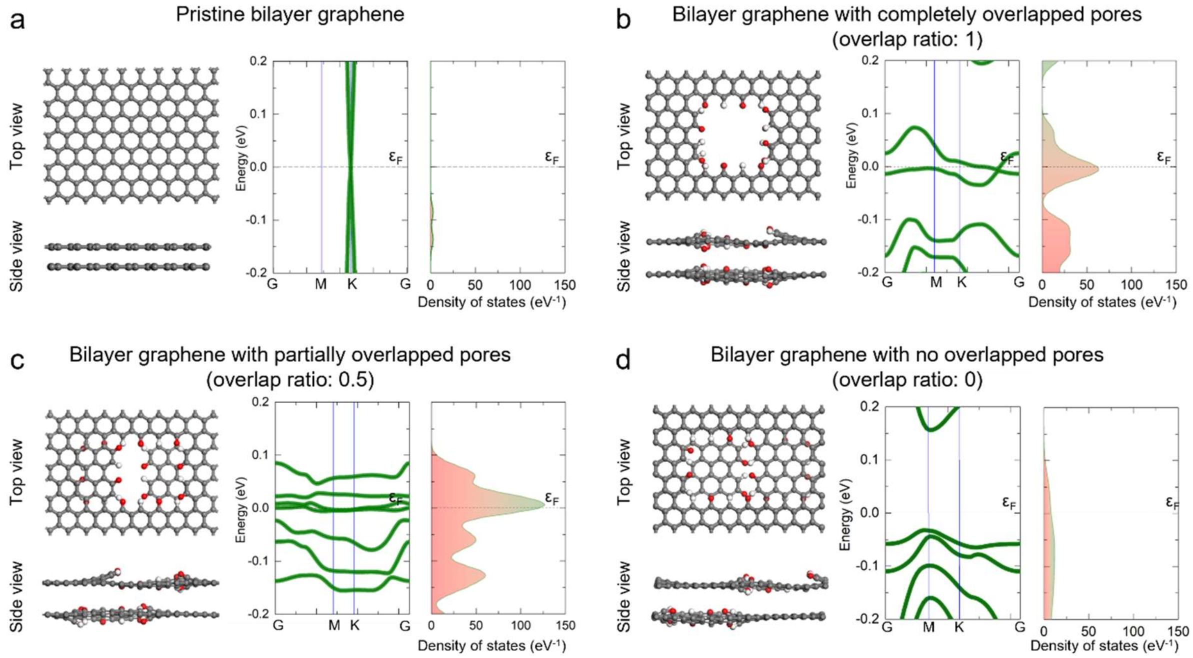

Supplementary Fig. 12 – Density functional theory (DFT) simulation of the band structure and projected density of states of graphene with different pore overlap ratios. a, Pristine graphene. b-d, Bilayer graphene with completely overlapped pores with an overlap ratio of 1, partially overlapped pores with an overlap ratio of 0.5, and no overlapped pores with an overlap ratio of 0. Zero density of states (ε_F) are indicated by the horizontal black dashed line. The size of the nanopores is 1.0 nm.

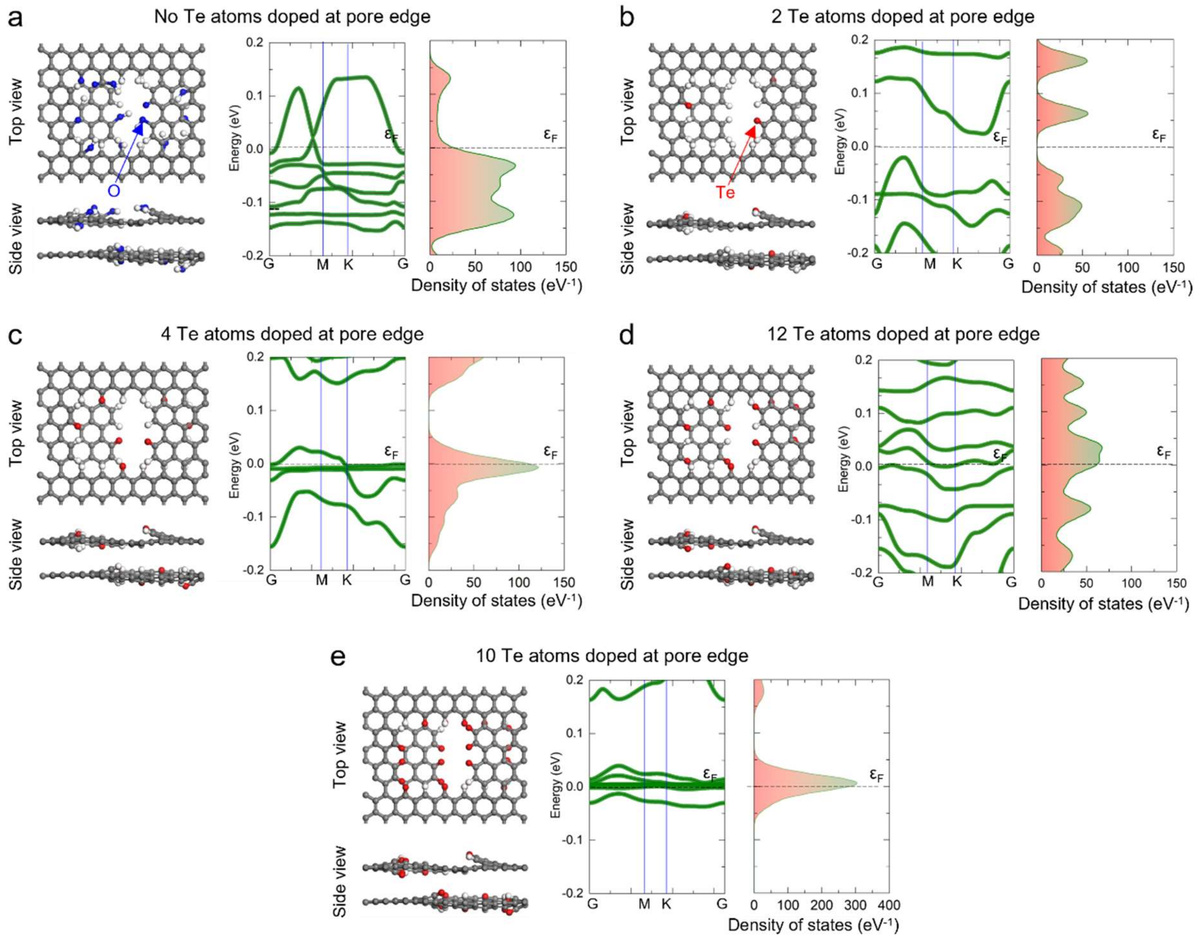

Supplementary Fig. 13 – DFT simulation of the band structure and projected density of states of graphene superlattice with varying Te doping levels. a-e, Graphene superlattice (a) without Te doping (carbon atoms at the pore edge were saturated with –H, –OH, =O and –O–) and with (b) two, (c) four, (d) six and (e) ten carbon atoms at the pore edge bonded with Te atoms for one layer. Zero density of states (ε_F) are indicated by the horizontal black dashed line. The size of the nanopores is 1.0 nm.

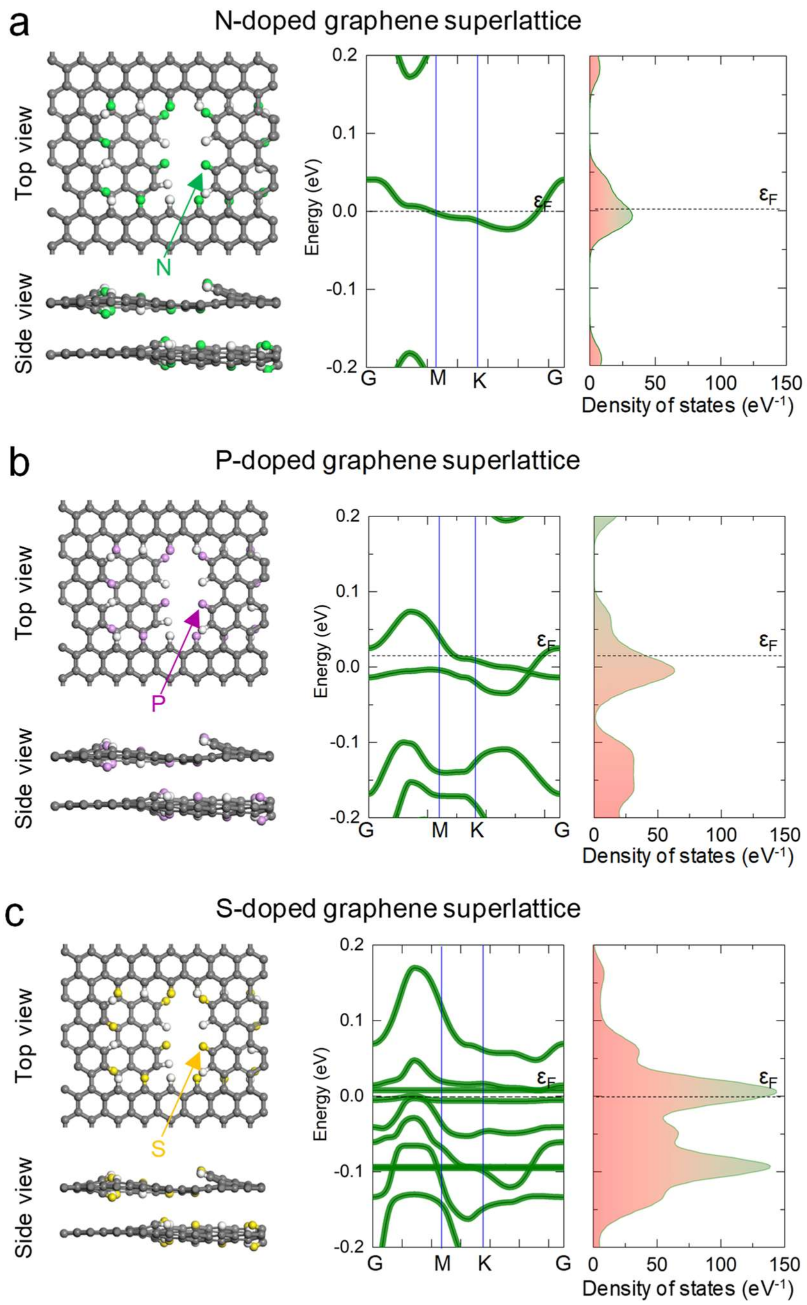

Supplementary Fig. 14 – DFT simulation of the band structure and projected density of states of graphene superlattice with various types of dopant elements. a-c, Graphene superlattice doped with N, P, and S. Eight carbon atoms at the pore edge were bonded with the above-mentioned atoms for one layer, while the remaining carbon atoms at the pore edge were saturated with H atoms. Zero density of states (ε_F) are indicated by the horizontal black dashed line. The size of the nanopores is 1.0 nm.

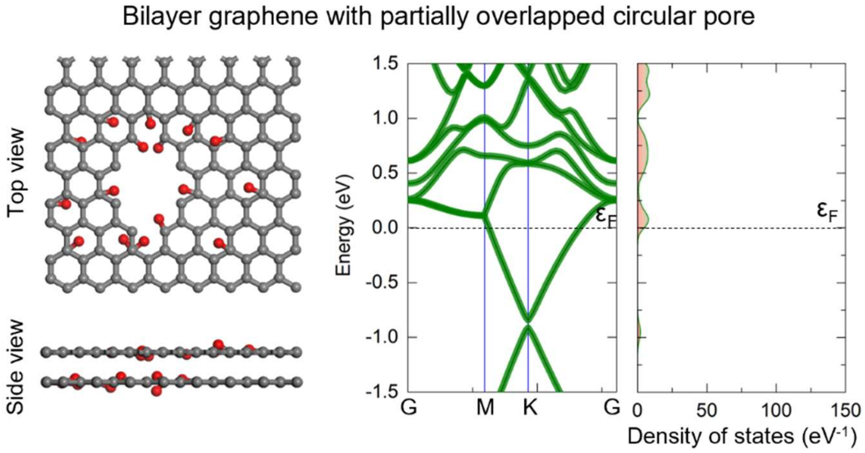

Supplementary Fig. 15 – DFT simulation of the band structure and projected density of states of bilayer graphene with circular pores. The overlap ratio is 0.5. Eight carbon atoms at the pore edge were bonded with Te for one layer, while the remaining carbon atoms at the pore edge were saturated with H atoms. Zero density of states are indicated by the horizontal black dashed line. The diameter of the nanopores is 1.0 nm.

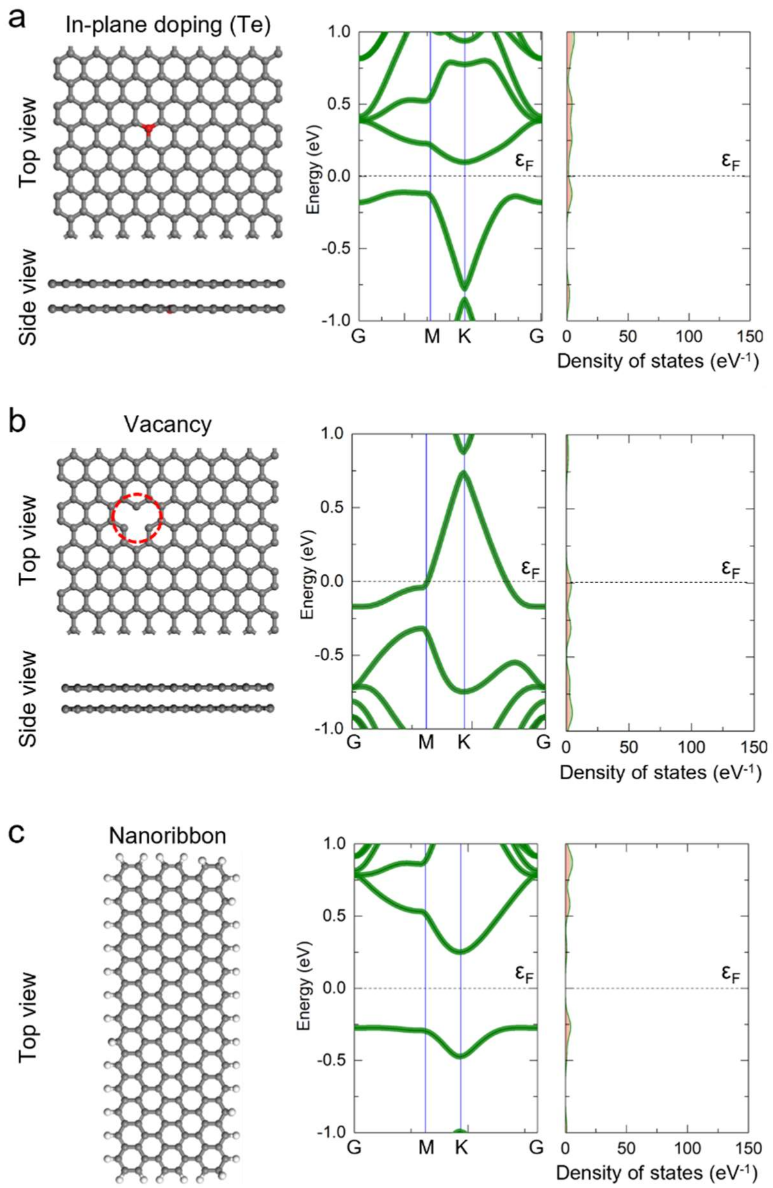

Supplementary Fig. 16 – DFT simulation of the band structure and projected density of states of graphene with varying defect structure. a-c, Te-doped pristine graphene, graphene with vacancy defects (i.e., a removed carbon atom), and an armchair graphene nanoribbon with 3.2 nm in length and 1.1 nm in width. The edges of the graphene nanoribbon were saturated with H atoms.

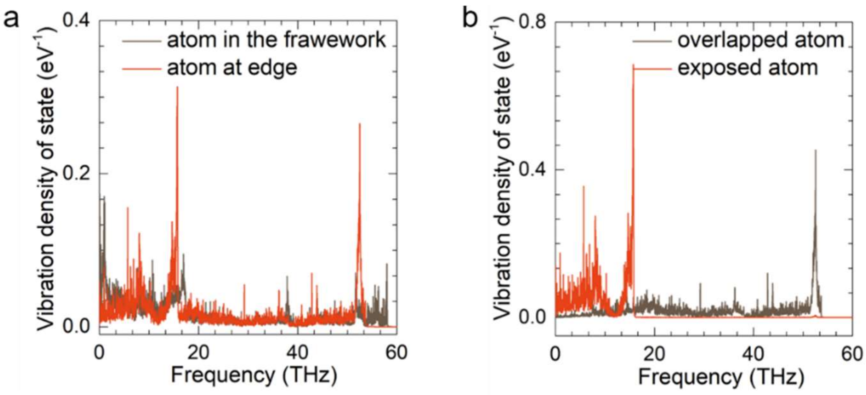

Supplementary Fig. 17 – DFT simulation of the vibrational density of states in Te-doped graphene superlattice. a, Vibrational density of states of carbon atom in the framework and at the pore edge. **b,** Vibrational density of states of overlapped carbon atoms and exposed carbon atoms.

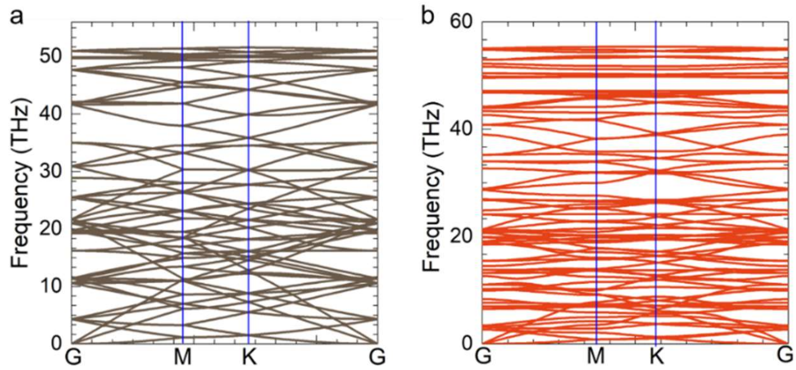

Supplementary Fig. 18 – DFT simulation of the phonon band structure of graphene. a, Pristine bilayer graphene. **b,** Te-doped graphene superlattice.

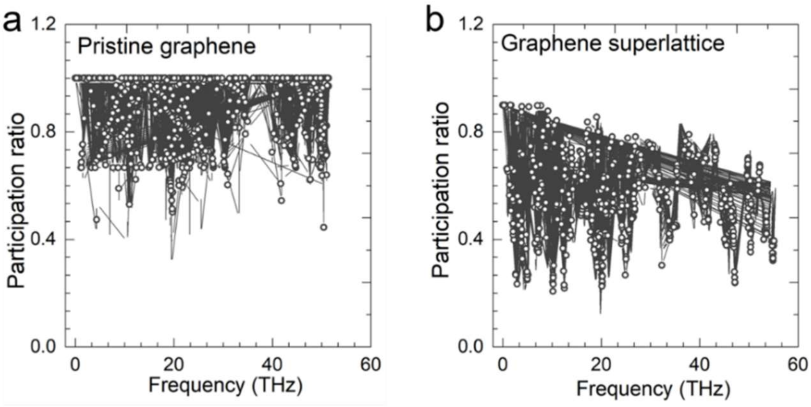

Supplementary Fig. 19 – DFT simulation of the phonon participation ratio of graphene. a, Pristine bilayer graphene. **b,** Te-doped graphene superlattice. The reduction in phonon energy stems from a decrease in the atomic participation ratio (PR), which can be calculated by $\mathrm{PR}_{\lambda}^{-1}=n\sum_{j}\left(\sum_{\alpha}\varepsilon_{j\partial,\lambda}^{*}\varepsilon_{j\alpha,\lambda}\right)^{2}$ [26], where n is the total number of atoms, j sums over all atoms, ∂ denotes a Cartesian direction, $\varepsilon^{*}_{j\alpha,\lambda}$ and $\varepsilon_{j\alpha,\lambda}$ represent the conjugated vibrational eigenvector component corresponding to the λ-th normal mode[81].

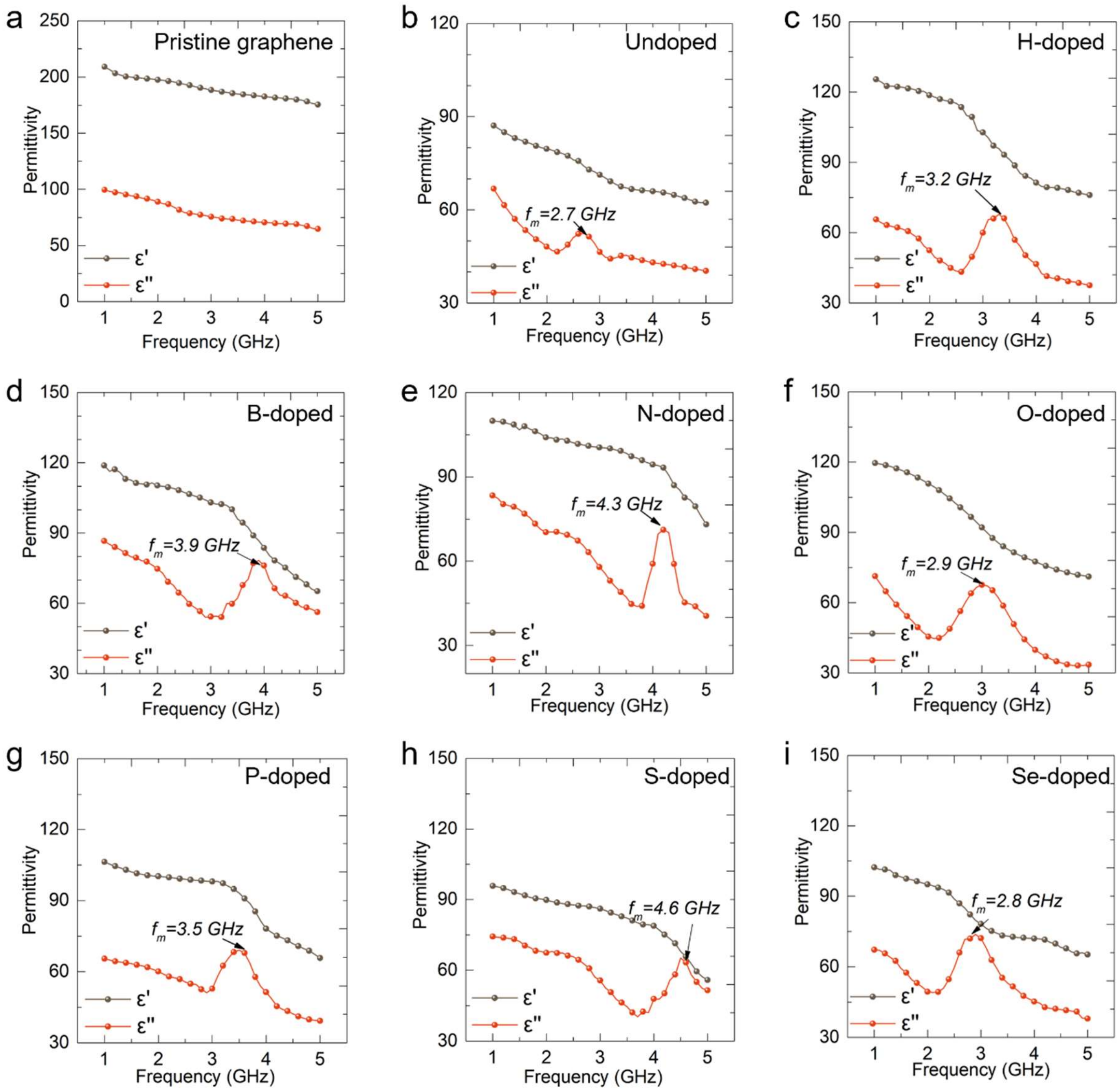

Supplementary Fig. 20 – Frequency-dependent permittivity of pristine graphene and graphene superlattice. a, Pristine bilayer graphene. **b,** Undoped porous graphene superlattice. **c-i,** Doped porous graphene superlattice. We observed dielectric relaxation polarization peaks in the low-frequency range for undoped porous graphene. However, it is evident that the polarization strength is considerably lower than that of the doped graphene superlattice. This discrepancy is attributed to the existence of various weak polarity bonds, consisting of a mixture of carbon, oxygen, and hydrogen, at the pore edges.

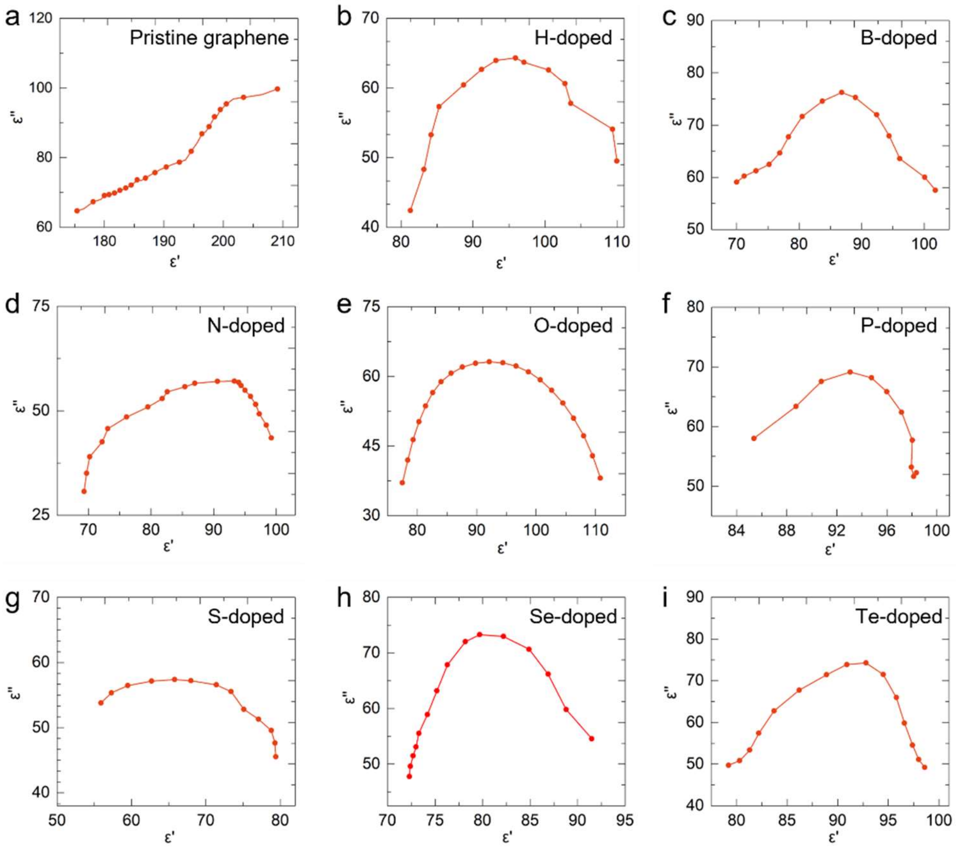

Supplementary Fig. 21 – Cole–Cole curves of pristine graphene and graphene superlattice. a, Pristine bilayer graphene. **b-i,** Graphene superlattice doped with H, B, N, O, P, S, Se, and Te. We observed that undoped porous graphene exhibits dielectric relaxation polarization peaks in the low-frequency range; however, it is evident that the polarization strength is significantly lower than that of the doped graphene superlattice. This is attributed to the presence of multiple types of weak polarity bonds composed of a mixture of carbon, oxygen, and hydrogen at the pore edges.

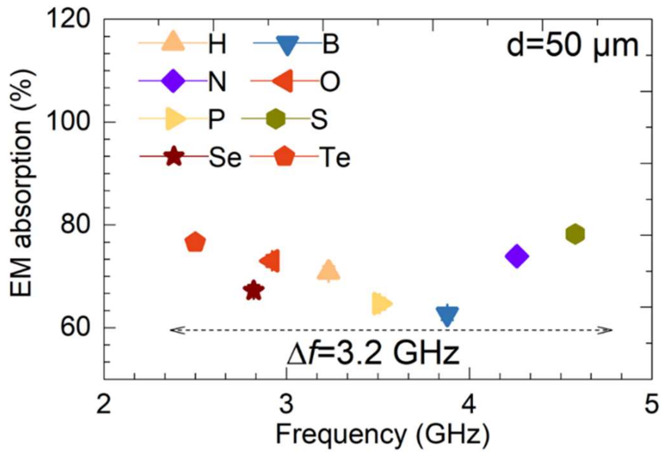

Supplementary Fig. 22 – Doped element-dependent EM absorption of graphene superlattice. The maximum absorption frequencies for different dopants in graphene superlattice were determined as follows: H-doped (3.2 GHz), B-doped (3.9 GHz), N-doped (4.3 GHz), S-doped (2.9 GHz), O-doped (3.5 GHz), P-doped (4.6 GHz), and Se-doped (2.8 GHz). Notably, all of these dopants exhibited EM absorption efficiencies exceeding 60%. This remarkable feature enables the graphene superlattice to selectively absorb EM waves at a specific wavelength while allowing the transmission of other frequency bands, thereby minimizing unnecessary signal loss. Error bars represent standard deviations from three independent measurements.

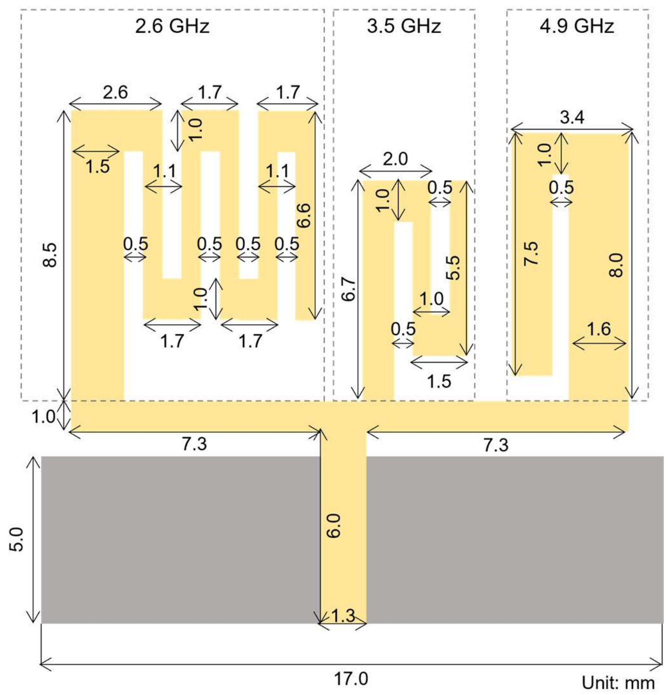

Supplementary Fig. 23 – Dimensions of graphene superlattice-based device designed for absorbing low-frequency EM waves. The optimal geometric shape, which allows for absorption in multiple lower-frequency bands, was determined using Computer Simulation Technology Microwave Studio software.

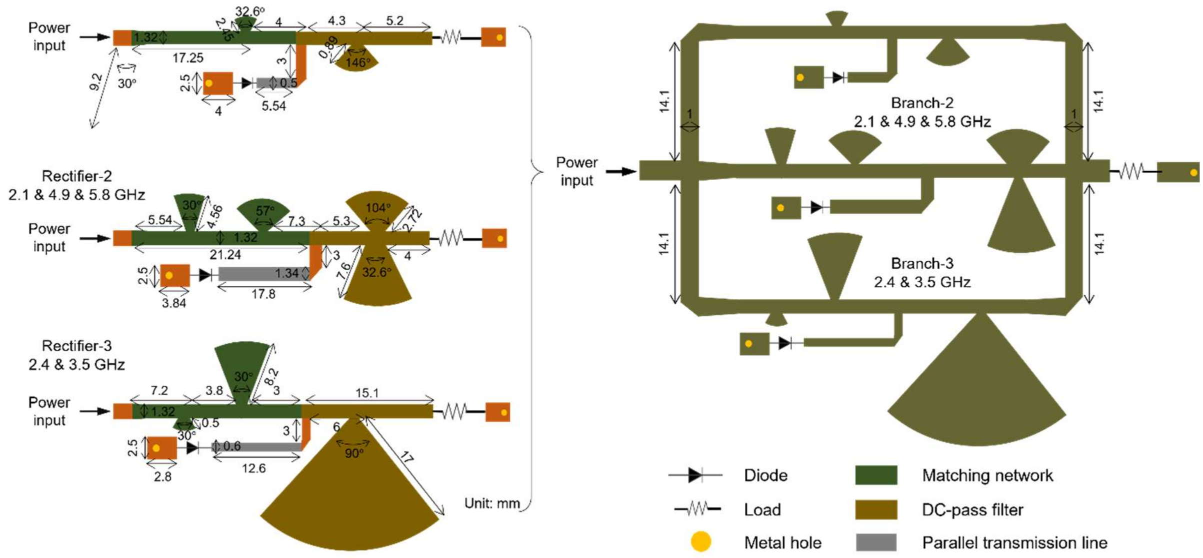

Supplementary Fig. 24 – Layout of rectifier for multiple low-frequency bands. The proposed layout for accommodating multiple low-frequency bands involves a rectifier with three parallel rectification branches. Each branch is constructed with a series connection of a diode, a DC-pass filter, a diode front-end transmission line, and an impedance matching network. The specific dimensions and geometric shapes of the rectifier are determined through calculations using the Advanced Design System software.

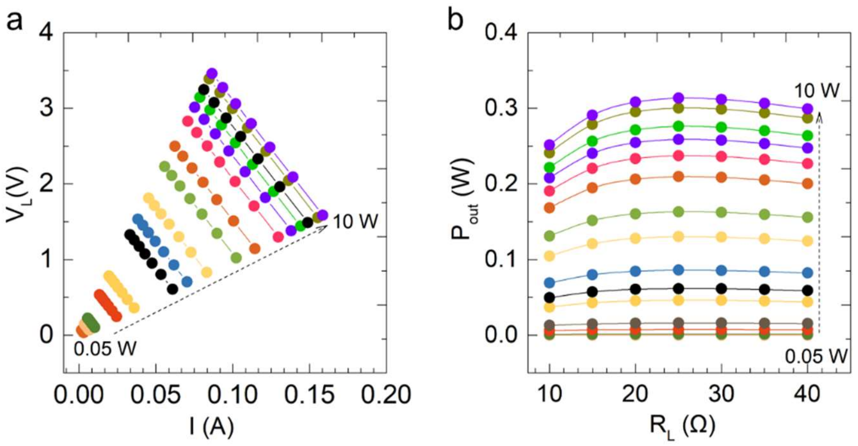

Supplementary Fig. 25 – Output voltage and output power of graphene superlattice-based device upon EM radiation. a, Output voltage (V_L)–current (I) curve of Te-doped graphene superlattice-based device with a load resistance ranging from 0 Ω to 40 Ω. **b,** Output power (P_{out}) of Te-doped graphene superlattice-based device as a function of load resistance, under different levels of EM wave radiation power (ranging from 0.05 W to 10 W).

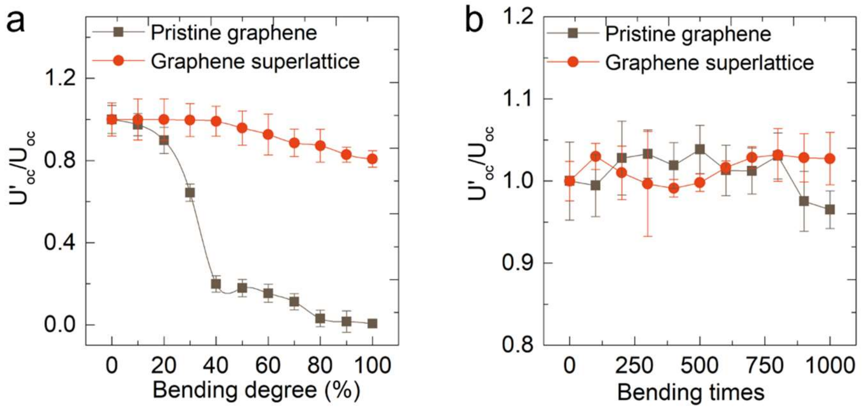

Supplementary Fig. 26 – Open circuit voltage of pristine graphene and graphene superlattice-based device after bending. a, b, The open circuit voltage of the Te-doped graphene superlattice-based device before (U_{oc}) and after (U'_{oc}) after subjecting it to different degrees of bending and varying bending cycles. Error bars represent standard deviations from three independent measurements on the same physical samples.

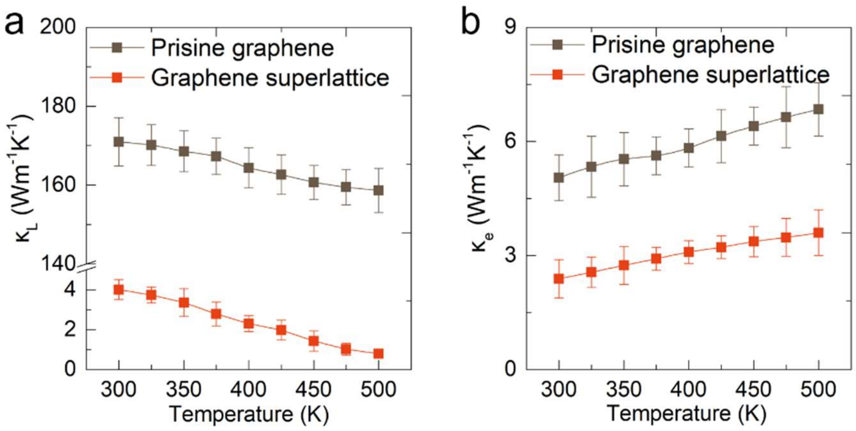

Supplementary Fig. 27 – Temperature-dependent in-plane thermal conductivity of pristine graphene and graphene superlattice film. a, lattice thermal conductivity. **b,** electron thermal conductivity. Error bars represent standard deviations from three independent measurements on the same physical samples.

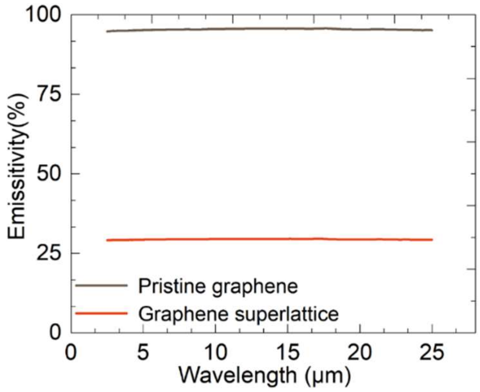

Supplementary Fig. 28 – Infrared emissivity of pristine graphene and graphene superlattice as a function of infrared wavelength. The results show that the Te-doped graphene superlattice exhibits an infrared emissivity of ~ 30%. This value was significantly lower compared to the infrared emissivity of pristine graphene. The lower infrared emissivity of the Te-doped graphene superlattice suggests that it has enhanced stealth capabilities in the infrared range.

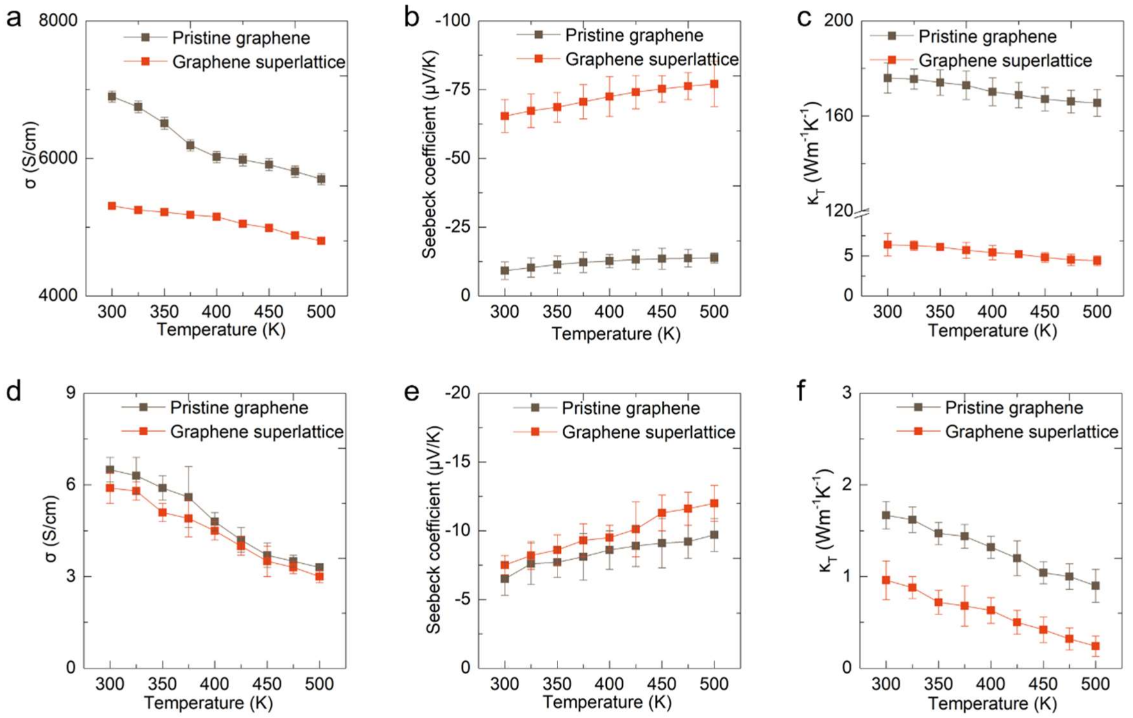

Supplementary Fig. 29 – Electrical and thermal conductivity of pristine graphene and graphene superlattice. a, In-plane electrical conductivity. **b,** In-plane Seebeck coefficient. **c,** In-plane thermal conductivity. **d,** Out-of-plane electrical conductivity. **e,** Out-of-plane Seebeck coefficient. **f,** Out-of-plane thermal conductivity. Error bars represent standard deviations from three independent measurements on the same physical samples.

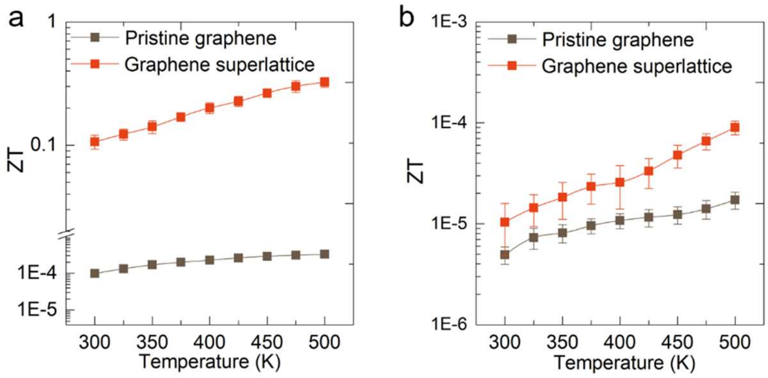

Supplementary Fig. 30 – Thermoelectric properties of pristine graphene and graphene superlattice. a, b, Figure of merit (*ZT*) values for (a) in-plane and (b) out-of-plane directions in graphene superlattice and pristine graphene. Error bars represent standard deviations from three independent measurements on the same physical samples.

Supplementary Tables

Supplementary Table 1. Polarization frequency, maximum EM absorption efficiency, and thickness of conventional dielectric materials.

Materials	Polarization relaxation (GHz)	A_{max} (%)	Thickness (μm)	References
Reduced graphene oxide (rGO)	> 12	< 10	1,500	51
I-doped graphene	> 18	< 1	12.5	52
S-doped graphene	> 18	~ 12.5	140	53
N-doped graphene	> 18	< 1	3	54
Lattice defect rGO	~ 11	< 5	1,000	55
Graphene oxide (GO)	> 18	< 5	11.8	56
Graphene microtube	> 18	~ 10	1,500	57
Graphene aerogel	> 18	~ 12	2,500	58
Graphene nanohybrids	> 12	< 5	1,500	59
Graphene foam	> 18	< 10	3,000	60
Multiwall carbon nanotubes	> 18	< 5	2,000	61
Carbon fibers	> 18	< 10	2,000	62
$Ti_3C_2T_x$	> 18	< 1	2.5	63
Ti_3CNT_x	> 18	< 1	40	64
Micropatternable MXene film	> 18	< 1	3.3	65
Ti_2CT_x aerogel	> 18	< 5	1,000	66
V_2CT_x	> 18	< 1	3,300	67
$Mo_2Ti_2C_3T_x$	> 18	< 5	5	68
$Ti_3C_2T_x$ film	> 18	< 1	~ 1	69
Porous $T_3C_2T_x$	> 18	< 10	1,900	70
Ti_3C_2 MXene/graphene	> 18	< 10	1,850	71
$Ti_3C_2T_x$/CNTs	> 18	< 10	38	72
$Ti_3C_2T_x$/BN	> 18	< 1	1,000	73
Porous $Ti_3C_2T_x$/rGO	> 18	< 1	1,500	74
$Ti_3C_2T_x$/C	> 18	< 15	2,000	75

Supplementary Table 2. Annealing treatment conditions for element doping.

Precursor	Mass (mg) or standard cubic Centimeters per minute (sccm)	Temperature (°C)	Time (h)	Atmosphere
B_2O_3	50 mg	1,150	1	Ar
NH_3	20 sccm	1,050	2	NH_3/Ar (1:5)
$BminPF_6$	45 mg	550	0.75	Ar
S powder	80 mg	500	0.5	Ar
Te powder	100 mg	650	0.5	Ar
Se powder	100 mg	650	0.5	Ar

Supplementary References

1. Wadey, J. D. *et al.* Mechanism of monovacancy diffusion in graphene. *Chem. Phys. Lett.* **648**, 161 (2016).
2. He, Y. Y. *et al.* A first-principles study of the effect of surface oxygen during the early stage of graphene growth in a Cu (111) surface. *Comput. Mater. Sci.* **168**, 17 (2017).
3. Qin, W. et al. Lithium diffusion in silicon encapsulated with graphene. *Nanomaterials* **11**, 3397 (2021).
4. Terrett, R. *et al.* Towards a computational understanding of water oxidation at graphene-bound Mn_xO_y and $Mn_xO_yM^{2+}$ particles. *Sustain. Energy Fuels.* **6**, 2276 (2022).
5. Peng, T. et al. Stacking stability of MoS_2 bilayer: An ab initio study. *Chin. Phys. B* **23**, 106801 (2014).
6. Yao, Y.G. *et al.* High temperature shockwave stabilized single atoms. *Nat. Nanotechnol.***14**, 851 (2019).
7. Fornasini, P. *et al.* Local structural distortions in SnTe investigated by EXAFS. *J. Phys-Condenes Mat.* **33**, 295404 (2021).
8. Caranazza, S. *et al.* Temperature dependent EXAFS study on transition metal dichalcogenides MoX_2 (X=S, Se, Te). *J. Phys-Condenes Mat.* **28**, 325401 (2016).
9. Sushkevich, V. *et al.* Structure of copper sites in zeolites examined by Fourier and wavelet transform analysis of EXAFS. *Chem Sci.* **11**, 5299 (2020).
10. Firet, N. *et al.* Operando EXAFS study reveals presence of oxygen in oxide-derived silver catalysts for electrochemical CO_2 reduction. *J. Mater. Chem. A* **7**, 2507 (2019).
11. Minasian, S. G. *et al.* New evidence for 5f covalency in actinosteles determined from carbon K-edge XAS and electronic structure theory. *Chem. Sci.* **5**, 351 (2014).
12. Povia, P. *et al.* Combining SAXS and XAS study the operando degradation of carbon-supported Pt-nanoparticle fuel cell catalysts. *ACS Catal.* **8**, 7000 (2018).
13. Zhong, J. *et al.* Synchrotron soft X-ray absorption spectroscopy study of carbon and silicon nanostructures for energy applications. *Adv. Mater.* **26**, 7786 (2014).
14. Hsu, C. H. *et al.* Description of photodegradation mechanisms and structural characteristics in carbon@titania yolk-shell nanostructures by XAS. *Small* **19**, 2203811 (2023).
15. Svintsiskiy, D. A. *et al.* Spectroscopic study of nitrogen distribution in N-doped carbon nanotubes and nanofibers synthesized by catalytic ethylene-ammonia decomposition. *Appl. Surf. Sci.* **435**, 1273 (2018).
16. Wu, B. *et al.* In -situ absorption spectroscopy of metal/nitrogen-doped carbons in oxygen electrocatalysis. *Angew. Int. Ed. Chem.* **13**, e202219188 (2023).
17. Bersha, A. *et al.* Interactions between the aryldiazonium cations and graphene oxide: A DFT study. *J. Chem.* **2**, 5126071 (2019).
18. Reda, M. *et al.* DFT study of stabilization effects on N-doped graphene for ORR catalysis. *Catal. Today* **312**, 118 (2018).
19. Deng, Q. *et al.* Electronic properties of triangle-shaped graphene nanoflakes from TAO-DFT. *ACS*

Omega **4**, 142020 (2019).
20. Raju, N. *et al.* Flat bands in twisted double bilayer graphene. *Phys. Rev. B.* **99**, 235417 (2019).
21. Mao, J. H. *et al.* Evidence of flat bands and correlated states in buckled graphene superlattices. *Nature* **215**, 584 (2020).
22. Roy, B. *et al.* Unconventional superconductivity in nearly flat bands in twisted bilayer graphene. *Phys. Rev. B.* **99**, 121407 (2019).
23. Wu, S. *et al.* Chern insulators, van Hove singularities and topological flat bands in magic-angle twisted bilayer graphene. *Nat. Mater.* **20**, 488 (2021).
24. Wolf, T. M. R. *et al.* Electrically tunable flat bands and magnetic in twisted bilayer graphene. *Phys. Rev. Lett*. **123**, 096802 (2019).
25. Ehlen, N. *et al.* Origin of the Flat band in heavily Cs-doped graphene. *ACS Nano* **14**, 1055 (2020).
26. Fedor, K. P. *et al*. Hidden wave function of twisted bilayer graphene: The flat band as a landau level. *Phys. Rev. Lett.* **103**, 155150 (2021).
27. Liu, J. P. *et al.* Orbital magnetic states in Moiré graphene systems. *Nat. Rev. Phys.* **3**. 367 (2021).
28. Garcia, J. H. *et al.* Spin transport in graphene/transition metal dichalcogenide heterostructures. *Chem. Soc. Rev*. **47**, 3359 (2018).
29. Zhou, X. *et al.* DFT study on the electronic structure and optical properties of N, Al and N-Al doped graphene. *Appl. Surf. Sci.* **450**, 354 (2018).
30. Goudarzi, M. *et al.* Electronic and optical properties of vacancy and B, N, O and F doped graphene: DFT study. *Opto-Electron. Rev.* **27**, 130 (2019).
31. Pawlak, R. *et al*. Bottom-up synthesis of Nitrogen-doped porous graphene nanoribbons. *J. Am. Chem. Soc.* **142**, 12568 (2020).
32. Singal, M. *et al.* Synergistic effect of Cu decoration and N doping in divacancy defected graphene nanoribbons on hydrogen gas sensing properties: DFT study. *Mater. Chem. Phys.* **273**, 125093 (2021).
33. Deji, R. *et al.* Density functional theory study of carbon nanoxide adsorption on transition metal doped armchair graphene nanoribbon. *Mater. Today Pro.* **54**, 771 (2022).
34. Gale, J. D. *et al.* A computer program fro the symmetry-adapted simulation of solids. *J. Chem. Soc.* **93**, 629 (1997).
35. Brenner, D. W. *et al.* Empirical potential for hydrocarbons for use in simulating the chemical vapor deposition of diamond films. *Phys. Rev. B* **42**, 9458 (1990).
36. Stuart, S. J. *et al.* Reactive potential for hydrocarbons with intermolecular interactions. *J. Chem. Phys.* **112**, 6472 (2000).
37. Kim. W. *et al.* Strategies for engineering in graphene nanomesh: Unraveling the role of Brillouin Zone folding, Phonon localization and phonon confinement. *Inter. J. Heat Mass Transfer.* **165**, 1982 (2021).
38. Luckyanova, M. N. *et al.* Phonon localization in heat conduction. *Sci. Adv.* **4**, 12 (2018).
39. Bodapati, A. *et al.* Vibrations and thermal transport in nanocrystalline silicon. *Phys. Rev. B* **74**, 24 (2006).

40. Wang, Y. *et al.* Edge effect on thermal transport in graphene nanoribbons: A phonon localization mechanism beyond edge roughness scattering. *Appl. Phys. Lett.* **1**, 101 (2012).
41. Guo, Z. X. *et al.* Thermal conductivity of graphene nanoribbons. *Appl. Phys. Lett.* **95**, 16 (2010).
42. Evans, W. J. *et al.* Thermal conductivity of graphene ribbons from equilibrium molecular dynamics: Effect of ribbon width, edge roughness, and hydrogen termination. *Appl. Phys. Lett.* **20**, 96 (2010).
43. Wu, Y. *et al.* Hierarchical construction of CNT networks in aramid papers for high-efficiency microwave absorption. *Nano. Res.* **16**, 7801 (2023).
44. Tao, J. Q. *et al.* Catfish effect induced by anion sequential doping for microwave absorption. *Adv. Funct. Mater.* **33**, 2211996 (2023).
45. Cheng, T. T. *et al*. Customizing the structure and chemical composition of ultralight carbon foams for superior microwave absorption performance. *Carbon* **206**, 181(2023).
46. Liu, Y. L. *et al.* Dual-pathway optimization on microwave absorption characteristics of core-shell Fe_3O_4@C microcapsules: Composition regulation on magnetic core and MoS_2 nanosheets growth on carbon shell. *Chem. Eng. J.* **461**, 141867 (2023).
47. Xiang, Z. N. *et al.* Microwave absorption performance of porous heterogeneous SiC/SiO_2 microspheres. *Chem. Eng. J.* **451**, 138742 (2023).
48. Wu, D. *et al.* Heterostructured CoFe@N-doped carbon porous polyhedron for efficient microwave absorption. *Nano Res.* **16**, 1859 (2023).
49. Liu, Y. J. *et al.* Multifunctional shape memory composites for Joule heating, self-healing, and highly efficient microwave absorption. *Adv. Funct. Mater.* **33**, 2211352 (2023).
50. Liu, M. *et al.* An ion-engineering strategy to design hollow FeCo/$CoFe_2O_4$ microspheres for high-performance microwave absorption. *Small* **19**, 2300363 (2023).
51. Wen, B. *et al.* Reduced graphene oxides: light-weight and high-efficiency electromagnetic interference shielding at elevated temperature. *Adv. Mater.* **26**, 3484 (2014).
52. Wang, Y. J. *et al.* Graphene paper for exceptional EMI shielding performance using large-sized graphene oxide sheets and doping strategy. *Carbon* **122**, 74 (2017).
53. Shahzad, F. *et al.* Sulfur-doped graphene laminates for EMI shielding applications. *J. Mater. Chem. C* **3**, 9802 (2015).
54. Lin. S. F. *et al.* Ultrathin nitrogen-doping graphene films for flexible and stretchable EMI shielding materials. *J. Mater. Sci.* **54**, 7165 (2019).
55. Kuang, B. Y. *et al.* Chemical reduction dependent dielectric and dielectric loss mechanism of reduced oxide. *Carbon* **127**, 209 (2018).
56. Shen, B. *et al.* Ultrathin flexible graphene films: an excellent thermal conducting materials with efficient EMI shielding. *Adv. Funct. Mater.* **24**, 4542 (2014).
57. Yin, X. M. *et al.* Lightweight and flexible 3D graphene microtubes membrane for high-efficiency electromagnetic-interference shielding. *Chem. Eng. J.* **387**, 124025 (2020).
58. Li, C. B. *et al.* Electromagnetic interference shielding of graphene aerogel with layered microstructure fabricated via mechanical compression. *ACS Appl. Mater. Interfaces* **12**, 30686 (2020).

59. Cao, M.S. *et al.* Graphene nanohybrids: excellent electromagnetic properties for the absorbing and shielding of electromagnetic waves. *J. Mater. Chem. C* **6**, 4586 (2018).
60. Li, J. C. *et al*. Bubble-templated rGO-graphene nanoplatelet foams encapsulated in silicon rubber for electromagnetic shielding and high thermal conductivity. *Chem. Eng. J.* **415**, 129054 (2021).
61. Guan, Q. F. *et al.* Sustainable double-network structural materials for electromagnetic shielding. *Nano Lett.* **21**, 2532 (2021).
62. Liang, J. Y. *et al.* Electromagnetic shielding property of carbon fiber felt made of different types of short-chopped carbon fibers. *Compos. Part A-Appl. S* **121**, 289 (2019).
63. Shahzad, F.*et al*. Electromagnetic interference shielding with 2D transition metal carbides (MXenes). *Science* **353**, 1137 (2016).
64. Iqbal, A. *et al.* Anomalous absorption of electromagnetic waves by 2D transition metal carbonitride Ti_3CNT_x (MXene). *Science* **369**, 446 (2020).
65. Lipton, J. *et al.* Scalable, highly conductive, and micropatternable MXene films for enhanced electromagnetic interference shielding. *Matter* **3**, 546 (2020).
66. Han, M. K. *et al.* Anisotropic MXene aerogels with a mechanically tunable ratio of electromagnetic wave reflection to absorption. *Adv. Opt. Mater.* **7**, 1900267 (2019).
67. Han, M. K. *et al.* Efficient microwave absorption with $V_{n+1}C_nT_x$ MXenes. *Cell Rep. Phys. Sci.* **10**, 101073 (2022).
68. Han, M. K. *et al.* Beyond $Ti_3C_2T_x$: MXenes for electromagnetic interference shielding. *ACS Nano* **14**, 5008 (2020).
69. Yun, T. *et al.* Electromagnetic shielding of monolayer MXene assemblies. *Adv. Mater*. **32**, 1906769 (2020).
70. Lu, Z.Q. *et al.* Micro-porous MXene/aramid nanofibers hybrid aerogel with reversible compression and efficient EMI shielding performance. *Compos. Part B-Eng.* **217**, 108853 (2021).
71. Tan, X. *et al.* Enhanced electromagnetic shielding and thermal conductive properties of polyolefin composites with a $Ti_3C_2T_x$ MXene/graphene framework connected by a hydrogen-bonded interface. *ACS Nano* **6**, 9254 (2022).
72. Cao, W. T. *et al.* Ultrathin and flexible CNTs/MXene/Cellulose nanofibrils composite paper for electromagnetic interface shielding. *Nano-Micro Lett.* **11**, 72 (2019).
73. Shang, Y. *et al.* Sandwiched cellulose nanofiber/boron nitride nanosheet/$Ti_3C_2T_x$ MXene composite film with high electromagnetic shielding and thermal conductivity yet insulation performance. *Compos. Sci. Technol.* **214**, 108974 (2021).
74. Zhang, Y. *et al.* Strong and conductive reduced graphene oxide-MXene porous films for efficient electromagnetic interference shielding. *Nano Res.* **15**, 4916 (2022).
75. Wang, L. *et al.* 3D $Ti_3C_2T_x$ MXene/C hybrid foam/epoxy nanocomposites with superior electromagnetic interference shielding performance and robust mechanical properties. *Compos. Part A-Appl. S.* **123**, 293 (2019).
76. Dragoman, M. *et al.* Nanomaterials and devices for harvesting ambient electromagnetic waves. *Nanomaterials* **13**, 595 (2023).

77. Aboualalaa, M. *et al.* Energy harvesting rectenna using high-gain triple-band antenna for powering internet-of-Things (IoT) devices in a smart office. *IEEE Trans Inst. Measurement* **72**, 2001313 (2023).
78. Lee, Y. C. *et al.* High-performance multiband ambient RF energy harvesting front-end system for sustainable IoT applications-review. *IEEE Access* **11**, 11143 (2023).
79. Mukerjee, M. *et al.* Recent advances in designing thermoelectric materials. *J. Mater. Chem. C* **35**, 12524 (2022).
80. Wang, S. *et al.* Progress of conjugated polymers as emerging thermoelectric materials. *Prog. Polym. Sci.* **129**, 101548 (2022).
81. Rahmani, H. *et al.* Next-generation IoT devices: Sustainable eco-friendly manufacturing, energy harvesting, and wireless connectivity. *IEEE J. Microwaves* **3**, 237 (2023).
82. Cui, L. et al. Thermal transport in graphene nanomesh: Unraveling the role of Brillouin zone folding, phonon localization and phonon confinement. *Int. J. Heat. Mass. Tran.* **165**, 120685 (2021).
83. Liu, J. *et al.* Hydrophobic, flexible, and lightweight MXene foams for high-performance electromagnetic-interference shielding. *Adv. Mater.* **29**, 1702367 (2017).
84. Rajavel, K. *et al*. Exfoliation and defect control of two-dimensional few-layer MXene $Ti_3C_2T_x$ for electromagnetic interference shielding coatings. *ACS Appl. Mater. Interfaces* **12**, 49737 (2020).
85. Yun, T. *et al*. Multidimensional $Ti_3C_2T_x$ MXene architectures via interfacial electrochemical self-assembly. *ACS Nano* **15**, 10058 (2021).
86. Shen, B. *et al*. Microcellular graphene foam for improved broadband electromagnetic interference shielding. *Carbon* **102**, 154 (2016).
87. Song, W. L. *et al.* Facile fabrication of ultrathin graphene papers for effective electromagnetic shielding. *J. Mater. Chem. C* **2**, 5057 (2014).
88. Li, C. B. *et al.* Electromagnetic interference shielding of graphene aerogel with layered microstructure fabricated via mechanical compression. *ACS Appl. Mater. Interfaces* **12**, 30686 (2020).
89. Shen, B. *et al.* Ultrathin flexible graphene film: an excellent thermal conducting material with efficient EMI shielding. *Adv. Funct. Mater.* **24**, 4542 (2014).
90. Wei, Q. *et al.* Superhigh electromagnetic interference shielding of ultrathin aligned pristine graphene nanosheets film. *Adv. Mater.* **34**, 1907411 (2020).
91. Kumar, P. *et al.* Large-area reduced graphene oxide thin film with excellent thermal conductivity and electromagnetic interference shielding effectiveness. *Carbon* **94**, 494 (2015).
92. Mirkhani, S. A. *et al.* Reduction of electrochemically exfoliated graphene films for high-performance electromagnetic interference shielding. *ACS Appl. Mater. Interfaces*, **13**, 15827 (2021).
93. Kashani, H. *et al*. Unprecedented electromagnetic interference shielding from three-dimensional bi-continuous nanoporous graphene. *Matter* **4**, 1077 (2019).
94. Liu, X. *et al*. Facile fabrication of ultrathin graphene film with ultrahigh electrical conductivity and superb electromagnetic interference shielding effectiveness. *J. Mater. Chem. C* **9**, 214 (2021).

95. Bi, S. *et al.* Electromagnetic interference shielding properties and mechanisms of chemically reduced graphene aerogels. *Appl. Surf. Sci.* **412**, 529 (2017).
96. Wang, Z. G. *et al.* Achieving excellent thermally conductive and electromagnetic shielding performance by nanostructure functionalization and oriented arrangement of carbon nanotubes in composite films. *Compos. Sci. Technol.* **194**, 108190 (2020).
97. Wang, C. *et al.* Direct growth of carbon nanotubes on basalt fiber for the application of electromagnetic interference shielding. *Carbon* **167**, 31 (2020).
98. He, W *et al.* High thermoelectric performance in low-cost $SnS_{0.91}Se_{0.09}$ crystals. *Science* **365**, 1418 (2019).
99. Zhou *et al.* Discovery of TaFeSb-based half-Heuslers with high thermoelectric performance. *Nat. Commun.* **10**, 270 (2019).
100. Luo, Z. Z. *et al*. Extraordinary role of Zn in enhancing thermoelectric performance of Ga-doped *n*-typed PbTe. *Energy Environ. Sci.* **15**, 368 (2022).
101. Moshwan, R. *et al.* Realizing high thermoelectric properties of SnTe via synergistic band engineering and structure engineering. *Nano Energy* **65**, 104056 (2019).
102. Ren G. K. *et al.* Complex electronic structure and compositing effect in high performance thermoelectric BiCuSeO. *Nat. Commun.* **10**, 2814 (2019).
103. Luo Y.B. *et al*. High thermoelectric performance in the new cubic semiconductor $AgSnSbSe_3$ by high-entropy engineering. *J. Am. Chem. Soc.* **142**, 15187 (2020).
104. Yang, S. Q. *et al.* Ductile $Ag_{20}S_7Te_3$ with excellent shape-conformability and high thermoelectric performance. *Adv. Mater.* **10**, 2007681 (2021).
105. Sarkar, D. *et al.* Metavalent bonding in GeSe leads to high thermoelectric performance. *Agnew Chem. Int. Ed.* **60**, 10350 (2021).
106. Jiang, Q. L. *et al.* High thermoelectric performance in n-type perylene bisimide induced by the Soret effect. *Adv. Mater.* **32**, 2002752 (2020)
107. Zheng, Z. H. *et al.* Rational band engineering and structural manipulations inducing high thermoelectric performance in *n*-type $CoSb_3$ thin films. *Nano Energy* **81**, 105683 (2021).
108. Yan X. M. *et al.* Melt-spun $Sn_{1-x-y}Sb_xMn_yTe$ with unique multiscale microstructures approaching exceptional average thermoelectric ZT. *Nano Energy* **84**, 105879 (2021).
109. Luo Y.B *et al.* High thermoelectric performance in the new cubic semiconductor $AgSnSbSe_3$ by high-entropy engineering. *J. Am. Chem. Soc.* **142**, 15187 (2020).
110. Xu, X. *et al.* Constructing van der Waals gaps in cubic-structured SnTe-based thermoelectric materials. *Energy Environ. Sci.* **12**, 5135 (2020).
111. Anno Y. *et al.* Enhancement of graphene thermoelectric performance through defect engineering. *2D Matter.* **4**, 0255019 (2017).
112. Stanley A. *et al.* Twisted grain boundary leads to high thermoelectric performance in tellurium crystals. *Energy Environ. Sci.* **16**, 2023 (2023).
113. Liu, Z.H. *et al.* Maximizing the performance of *n*-type Mg_3Bi_2 based materials for room-temperature power generation and thermoelectric cooling. *Nat. Commun.* **13**, 1120 (2022).

114. Yu, L.Q. *et al.* Dense dislocations enable high-performance PbSe thermoelectric at low-medium temperatures. *Nat. Commun.* **13**, 6449 (2022).

115. Zhu, T.J. *et al.* High efficiency Half-Heusler thermoelectric materials for energy harvesting. *Adv. Energy Mater.* **19**, 1500588 (2015).

116. Liu Z. H. *et al.* Design of high-performance disordered Half-Heusler thermoelectric materials using 18-electron rule. *Adv. Funct. Mater.* **29**, 1905044 (2019).

117. Jeong, H. *et al.* Origin of low thermal conductivity in $Nb_{1-x}Ti_xFe_{1.02}Sb$ half-Heusler thermoelectric materials. *J. Europ. Ceram. Soc.* **41**, 4175 (2021).

118. Li, X. F. *et al.* Phase boundary mapping in ZrNiSn Half-heusler for enhanced thermoelectric performance. *Research* **2**, 4630948 (2020).

119. Li, W. J. *et al.* Enhanced thermoelectric performance of Yb-single-filled Skutterudite by ultralow thermal conductivity. *Chem. Mater.* **31**, 862 (2019).

120. Liu, Z. Y. *et al.* High-performance *n*-type $CoSb_3$-based thermoelectric material with vortex and strip-shaped grain structures. *J. Europ. Ceram. Soc.* **43**, 1985 (2023).

121. Fan, X. *et al.* $S_{0.05}Co_4Sb_{11.6}Te_{0.4}$ skutterudite introduced graphene at high pressure and high temperature and its thermoelectric performance enhancement. *Ceram. Int.* **48**, 15136 (2022).

122. He, W.K. *et al.* High thermoelectric performance in low-cost $SnS_{0.91}Se_{0.09}$ crystals. *Science* **365**, 1418 (2019).

123. Xiao, Y. *et al.* Ultrahigh carrier mobility contributes to remarkably enhanced thermoelectric performance in *n*-type PbSe. *Energy Environ. Sci.* **15**, 346 (2022).

124. Chen, Z. X. *et al.* GaSb doping facilitates conduction band convergence and improves thermoelectric performance in *n*-type PbS. *Energy Environ. Sci.* **16**, 1676 (2023).

125. Chen, C. *et al.* Zintl-phase Eu_2ZnSb_2:a promising thermoelectric materials with ultralow thermal conductivity. *Proc. Natl. Acad. Sci.* **116**, 2831 (2019).

126. Subhajit, R. *et al.* Enhanced atomic ordering leads to high thermoelectric performance in $AgSbTe_2$. *Science* **371**, 722 (2021).

Note: References 82-96 cited in **Fig. 3c** of the main text and References 97-126 cited in **Fig. 4e** of the main text.
