# Supplementary material for: Functional nanoporous graphene superlattice
Source: Nat Commun. 2024 Feb 12;15:1295. doi: 10.1038/s41467-024-45503-9 (PMC10861524; doi:10.1038/s41467-024-45503-9)
Supplement: Supplementary file 3 — Description of Additional Supplementary Files [file 41467_2024_45503_MOESM3_ESM.pdf]

Description of Additional Supplementary Files

File Name: Supplementary Movie 1
Description: Harvesting and converting electromagnetic waves from a cell phone into direct current electricity. During a phone call, the electromagnetic signal near an iPhone 12 experiences a significant enhancement for improved reception. The graphene superlattice-based device efficiently harvests the excess electromagnetic signals and converts them into direct current (DC) electricity. The multimeter measures the output voltage of the graphene superlattice-based device.

File Name: Supplementary Movie 2
Description: Harvesting electromagnetic waves from a telecommunication tower and converting them into DC electricity. A graphene superlattice-based device positioned 500 meters away from a telecommunications tower effectively captures and harvests weak electromagnetic signals, converting them into DC electricity. The multimeter measures the output voltage of the graphene superlattice-based device.

File Name: Supplementary Movie 3
Description: Converting electromagnetic waves from a microwave oven into DC electricity to light up LEDs. When the Panasonic home microwave oven (Model: NN-GT353 M) operates at a power consumption of 800 W for 20 seconds, it generates electromagnetic wave leakage that radiates into the surrounding air. The graphene superlattice-based device efficiently captures these leaked electromagnetic waves and converts them into DC electricity, which is used to power an 'OSU' pattern of LEDs. The pattern consists of 26 LEDs connected in parallel, with each LED having a power consumption of 60 mW.

File Name: Supplementary Movie 4
Description: **Converting and storing electromagnetic waves from a Wi-Fi Router as DC electricity to power a digital hygro-thermometer.** By positioning a graphene superlattice-based device in proximity to a home Wi-Fi router, which emits electromagnetic energy with a power output of approximately 50 mW, the device harvests and converts the electromagnetic waves into DC electricity. The captured DC electricity can then be stored and utilized to power a Dretect digital hygro-thermometer with a power consumption of 100 mW after just one minute of storage. The multimeter measures the charging voltage of the hygro-thermometer.
